# Serum Myoglobin Is Associated With Postoperative Acute Kidney Injury in Stanford Type A Aortic Dissection

**DOI:** 10.3389/fmed.2022.821418

**Published:** 2022-02-22

**Authors:** Chen Yang, Peng Hou, Dongxu Wang, Zhenguo Wang, Weixun Duan, Jincheng Liu, Shiqiang Yu, Feng Fu, Zhenxiao Jin

**Affiliations:** ^1^Department of Cardiovascular Surgery, Xijing Hospital, Fourth Military Medical University, Xi'an, China; ^2^Department of Biomedical Engineering, Fourth Military Medical University, Xi'an, China

**Keywords:** aortic dissection, total aortic arch replacement, acute kidney injury, myoglobin, rhabdomyolysis

## Abstract

**Background:**

The correlation between rhabdomyolysis and postoperative acute kidney injury has been reported in several surgical procedures. As a good predictor of rhabdomyolysis-related acute kidney injury, an elevated serum myoglobin level was often observed after total aortic arch replacement combined with frozen elephant trunk implantation. However, the correlation between serum myoglobin and acute kidney injury in such patients had not been established.

**Methods:**

Totally 398 stanford type A aortic dissection patients who underwent total aortic arch replacement combined with frozen elephant trunk implantation were enrolled in this retrospective study. The correlations between serum myoglobin and acute kidney injury as well as the 30-day mortality were assessed.

**Results:**

Overall, 268(67.3%) patients had acute kidney injury (KDIGO stage 1 or higher) and 75(18.8%) had severe acute kidney injury (KDIGO stage 2&3). Patients who developed acute kidney injury had higher level of perioperative serum myoglobin than patients without acute kidney injury. After adjusting for known acute kidney injury risk factors, logarithmically transformed preoperative serum myoglobin [*OR* = 1.58 (95% CI, 1.26–1.95), *P* < 0.001] and postoperative day 1 serum myoglobin [*OR* = 3.47 (95%CI, 2.27–5.29), *P* < 0.001] were associated with severe acute kidney injury. These correlation persisted after adjustment for decline in filtration via change in serum creatinine (ΔCr) and biomarkers of cardiac and kidney injury, including N-terminal prohormone of brain natriuretic peptide, cardiac troponin I, creatine kinase-MB, serum creatinine and Cystatin C. Compared with the clinical model, sMb considerably improved the risk discrimination and reclassification for AKI.

**Conclusion:**

For stanford type A aortic dissection patients underwent total aortic arch replacement with frozen elephant trunk implantation, serum myoglobin can improve postoperative acute kidney injury risk classification. Rhabdomyolysis may be an important supplement to the existing knowledge on the mechanism of acute kidney injury.

## Introduction

Type A aortic dissection (TAAD) is a life-threatening cardiovascular disease. Total arch replacement (TAR) with the frozen elephant trunk (FET) implantation is an effective treatment for TAAD and produces favorable clinical outcomes (1). However, postoperative acute kidney injury (AKI) exhibits high morbidity (50–70%) and indexes poor prognosis (2, 3). Since the mechanism of AKI has not been fully clarified, researches on the relationship between postoperative AKI and biomarkers with different pathophysiological backgrounds received extensive attention. Furthermore, pathophysiology-guided strategies are expected to translate into improvements in AKI clinical prevention, diagnosis and therapy (4). Among them, the relevance of Cystatin C (CysC), a renal biomarker of glomerular filtration, with AKI has been demonstrated (5). Meanwhile, several studies have shown that cardiac injury biomarkers can improve risk discrimination and reclassification for AKI, including N-terminal prohormone of brain natriuretic peptide (NT-proBNP), creatine kinase-MB (CK-MB) (6, 7).

Myoglobin is a small-molecule oxygen-binding protein (18 kD) which is abundant in myocardial and skeletal muscle. However, sMb has been forsaken by cardiac troponin I (cTnI) for diagnosing myocardial injury (8). A recent study showed that myoglobin is an excellent skeletal muscle damage marker which could serve as an early diagnostic index for pressure induced deep tissue injury (9). Simultaneously, with the nephrotoxicity of myoglobin elucidated, the predictive value of serum myoglobin (sMb) for rhabdomyolysis-related AKI was reported in many surgical procedures (10–14). However, its correlation with AKI in the TAAD patients following TAR with FET had not been established. The purposes of this study were: (1) detect the relationship between sMb and postoperative AKI, (2) determine whether sMb level can improve risk prediction of AKI, and (3) screen the risk factors of elevated sMb.

## Materials and Methods

### Patients

This retrospective study was approved by the Ethics Committee of Xijing Hospital and the requirement for informed consent was waived off. This study was registered in the Chinese Clinical Trial Registry (ChiCTR2100042849).

From December 2017 to January 2020, 411 consecutive TAAD patients who underwent TAR with FET at Xijing Hospital. Exclusion criteria was the followings: (1) a history of end stage renal disease (ESRD) requiring dialysis; (2) pregnant women; (3) patients died within 48 h after surgery; (4) patients with incomplete data. Finally, a total of 398 patients were included in the analysis (Supplementary Figure 1). Clinical data were collected retrospectively from the clinical database of Xijing hospital.

### Sample Size Calculation

The risk of AKI after TAR with FET was estimated to be 60% based on the literatures (2, 3). The sample size calculation was intended to detect a proportion of 60% with a margin of error of 10% and a confidence interval of 95%, leading to a sample size calculation of 387 participants. Finally, 398 patients were included. Sample size calculation was determined with PASS 11.

### Surgical Technique

All study participants underwent TAR with FET implantation. This procedure integrates total arch replacement using 4-branch arch Gelweave graft (Vascutek Terumo Inc, Scotland, England) with implantation of a frozen elephant trunk (MicroPort Medical, Shanghai, China) (Supplementary Figure 2) in the descending aorta as the treatment for extensive dissections or aneurysms involving the ascending aorta, the aortic arch, and the descending aorta. Moderate hypothermic circulatory arrest (MHCA, 25–28°C) and selective cerebral perfusion (ACP, 5-10 mL/kg/min) were performed routinely in the procedure. Sufentanil, rocuronium, propofol and midazolam were administered intravenously at the beginning of anesthesia induction, and sufentanil, pipecuronium, midazolam were used for maintaining anesthesia during the operation. Postoperative analgesia was carried out with sufentanil and remifentanil. All surgical procedures were performed by the same surgical team.

### Biomarker Assays

Conventional serological tests of cardiac injury biomarkers (NT-proBNP, cTnI, CK-MB) and myoglobin were performed before surgery and on the 3 days after the operation. Serum CysC and creatinine were continuously monitored at least 7 days after surgery. The preoperative biomarkers were measured at induction of anesthesia. Creatinine kinase (CK) which failed to be proved as a reliable predictor of rhabdomyolysis-related AKI is not assayed (15). All laboratory assays were conducted in the Department of Laboratory Medicine of Xijing Hospital, using the commercial kits provided by the same company with the analyzers. CK-MB, cTnI and sMb were tested by BECKMAN DXI800 analyzer (Beckman Coulter, America). NT-proBNP tested by Cobas8000 C701 analyzer (Roche, Germany). H7180 analyzer (Hitachi, Japan) was used for CysC test.

### Definition

Postoperative AKI was defined according to the Kidney Disease Improving Global Outcomes (KDIGO) criteria (16). The primary outcomes were any AKI (KDIGO stage 1 or higher) and severe AKI (KDIGO stage 2 and 3). Any AKI corresponded to serum creatinine increase ≥26.5 μmol/L (within 48 h) or serum creatinine 1.5 times the baseline (within 7 days), while severe AKI corresponded to doubling of serum creatinine (within 7 days) or AKI requiring dialysis. Urine output was not used to evaluate the level of AKI in this study. Creatinine changes within 7 days after surgery was used to define AKI as most articles focused on the CSA-AKI (16–18). Therefore, the internationally recognized diagnostic criterion of AKI was applied to ensure clinical significance. The second outcome was 30-day mortality which were collected with clinical records review or telephone follow-up. Estimated glomerular filtration rate (eGFR) was calculated using the CKD Epidemiology Collaboration (CKD-EPI) formula (19). Oliguria was defined as a urine output <125 ml/6 h or 400 mL/24 h.

### Statistical Methods

All normally distributed continuous variables were described with means [±standard deviation (SD)] and compared across groups with Student's *t*-test. Median [interquartile range (IQR)] and the Wilcoxon rank sum test were used for non-normally distributed continuous variables. Categorical variables were described with frequencies (%) and compared with chi-square test or Fisher's exact test. NT-proBNP, cTnI, CK-MB, CysC, and sMb were transformed by natural logarithm due to skewed distributions. Multivariate logistic regression was used to predict the standardized odds ratios (OR) of AKI. Two models which include the important clinical covariates in common clinical prediction scores for AKI after cardiac surgery were constructed to adjust (20–22). The preoperative model (Model^a^) includes age, sex, body mass index (BMI), hypertension, preoperative eGFR, preoperative white blood cell (WBC) and preoperative lactate. In addition to all covariates of the Model^a^, the postoperative model (Model^b^) includes surgery duration, cardiopulmonary bypass (CPB) duration, MHCA temperature and lactate. Then, the ORs of AKI were further adjusted for changes in serum creatinine (the value at the same time point as sMb minus preoperative value) (ΔCr) and logarithmically transformed cardiac biomarkers and renal biomarker, including NT-proBNP, cTnI, CK-MB and CysC. The ability of the postoperative day 1 (POD1) sMb level to discriminate risk for AKI based on the Model^b^ was evaluated using area under the receiver operating characteristic curve (AUC), net reclassification improvement (NRI), and integrated discrimination improvement (IDI) (23). Pairwise comparison of AUC values was performed by DeLong test. In addition, receiver operating characteristic (ROC) curve analysis was performed to determine a cut-off value of the POD1 Ln(sMb) to predict AKI and severe AKI. We demonstrated the non-linear relationship between POD1 Ln(sMb) and severe AKI with adjustment for Model^b^ using restricted cubic spline. Furthermore, stepwise linear regression analysis was used to assess independent predictors for the POD1 Ln(sMb) levels. A *P*-value of <0.05 was considered statistically significant. Small amouts of missing values were statistically imputed using mean imputation. All analyses were performed with SPSS 19.0 and R (version 4.0.0.0).

## Results

### Patient Characteristics

Baseline characteristics of patients without AKI, with any AKI and with severe AKI are presented in Table 1. Overall, 268 patients (67.3%) experienced any AKI and 75 patients (18.8%) had severe AKI during hospital stay. 57 patients (14.3%) received renal replacement therapy (RRT) which includes 20 patients (5.0%) received RRT between POD1 and POD3. 30-day mortality was 11.8%. Notably, 60% of our patients are diagnosed in local hospitals and transferred to our institution. In this cohort of patients, 114 (28.6%) patients were operated in <24 h after event occurrence, 168 (42.2%) patients were operated in 24–48 h after event occurrence, 52 (13.1%) patients were operated in 48–72 h and 64 patients were operated in >72 h after event occurrence.

**Table 1:** Patient characteristics by different AKI severity.

**Characteristic**	**All (*n* = 398)**	**No AKI (*n* = 130)**	**Any AKI (*n* = 268)**	**Severe AKI**	***P*-value**	***P*-value**
				**(*n* = 75)**	**(Any AKI vs. NOT)^a^**	**(Severe AKI vs. NOT)^b^**
**Demographics**						
Gender (Male)	318 (79.9)	98 (75.4)	220 (82.1)	67 (89.3)	0.117	0.015
Age (y)	48.4 ± 9.8	47.2 ± 10.5	49.0 ± 9.4	47.0 ± 9.2	0.089	0.850
BMI (kg/m^2^)	25.3 ± 3.6	24.3 ± 3.5	25.8 ± 3.6	27.1 ± 3.9	<0.001	<0.001
**Comorbidities**						
Hypertension	295 (74.1)	91 (70.0)	204 (76.1)	52 (69.3)	0.191	0.920
Diabetes	5 (1.3)	0 (0)	5 (1.9)	2 (2.7)	0.178	0.133
CAD	9 (2.3)	2 (1.5)	7 (1.9)	1 (1.3)	0.724	1.000
Marfan syndrome	7 (1.8)	4 (3.1)	3 (1.1)	1 (1.3)	0.222	0.654
History of cardiac surgery	14 (3.5)	4 (3.1)	10 (3.7)	2 (2.2)	1.000	1.000
COPD	5 (1.3)	3 (2.3)	2 (0.7)	1 (1.5)	0.336	1.000
Iliac artery involvement	225 (56.5)	67 (51.5)	158 (58.9)	45 (60.0)	0.162	0.532
**Marker at baseline**						
WBC counts (10^9^/L)	11.3 (9.0, 14.1)	10.7 (8.3, 12.4)	11.7 (9.3, 14.6)	13.0 (9.4,15.4)	0.001	0.001
Cr (μmoI/L)	90 (70, 111)	88 (73, 108)	92 (75, 115)	98 (76, 134)	0.162	0.075
eGFR (ml/min/1.73m^2^)	78.8 (61.3, 99.1)	81.4 (63.6, 99.8)	77.2 (56.8,98.0)	76.3 (49.6, 102.0)	0.379	0.437
LVEF (%)	57 (54,60)	57 (53, 60)	58 (54, 61)	57 (53,59)	0.160	0.001
**Surgical characteristics**						
No-elective surgery	167 (42.0)	50 (38.5)	117 (43.7)	41 (54.7)	0.325	0.024
Femoral cannulation	207 (52.0)	64 (49.2)	143 (53.4)	47 (62.7)	0.440	0.040
Combined with Bentall	167 (42.0)	56 (43.1)	111 (41.4)	36 (48.0)	0.753	0.495
Combined with CABG	195 (49.0)	66 (50.8)	129 (48.1)	32 (42.7)	0.622	0.263
Operation time (min)	385 (349, 431)	365 (330, 405)	395 (355, 450)	415 (370, 485)	<0.001	0.001
CPB duration (min)	215 (195, 236)	201 (183, 227)	220 (200, 240)	230 (209, 252)	<0.001	0.001
ACC duration (min)	67 (84, 110)	93 (82, 107)	100 (84, 111)	99 (85, 118)	0.041	0.065
MHCA duration (min)	31 (27, 36)	31 (26, 35)	32 (27, 36)	33 (27, 37)	0.087	0.131
MHCA temperature (°C)	26.0 (25.6, 26.5)	26.0 (25.6, 26.3)	26.0 (25.6, 26.6)	26.0 (25.5, 26.7)	0.309	0.518
pRBC transfusion (U)	9.0 (5.0, 13.5)	7.0 (4.0, 10.5)	10.3 (6.0, 16.0)	14.0 (8.0,22.5)	<0.001	0.001
pH on ICU admission	7.42 ± 0.10	7.45 ± 0.09	7.41 ± 0.09	7.37 ± 0.08	<0.001	<0.001
**Postoperative complication**						
Ventilator duration (h)	21 (16, 46)	18 (16, 22)	37 (17, 59)	63 (37,88)	<0.001	0.001
ICU LOS (d)	3 (2,5)	2 (2, 3)	3 (2, 5)	6 (4,10)	<0.001	0.001
30-day mortality	47 (11.8)	1 (0.8)	46 (17.2)	35 (46.7)	<0.001	<0.001
Oliguria in POD1	16 (4.0)	5 (3.8)	11 (4.1)	4 (5.3)	0.902	0.520
Cardiovascular complications	56 (14.1)	5 (3.8)	51 (19.0)	32 (42.7)	<0.001	<0.001
Respiratory complications	60 (15.1)	18 (3.8)	42 (15.7)	14 (18.6)	0.460	0.268
Neurological complications	63 (15.8)	6 (4.6)	57 (21.3)	29 (38.7)	<0.001	<0.001
Hepatic dysfunction	38 (9.50)	4 (3.1)	34 (12.7)	23 (30.7)	0.002	<0.001
Sepsis	20 (5.0)	2 (1.5)	18 (6.7)	10 (13.3)	0.027	0.002
Re-exploration for bleeding	3 (0.8)	1 (0.8)	2 (0.7)	2 (1.1)	1.000	0.556

*BMI, Body mass index; CAD, Coronary artery disease; COPD, Chronic obstructive pulmonary disease; WBC, White blood cell; eGFR, estimated glomerular filtration rate; LVEF, Left ventricular ejection fraction; CPB, cardiopulmonary bypass; ACC, Aortic cross-clamp; MHCA, Moderate hypothermic circulatory arrest; pRBC, packed red blood cells; ICU LOS, ICU length of stay.*

*Any AKI is KDIGO stage 1 or higher. Severe AKI is KDIGO stage 2 and 3. All normally distributed continuous variables were described with means (±standard deviation [SD]) and compared across groups with Student t-test. Median (interquartile range [IQR]) and the Wilcoxon rank sum test were used for non-normally distributed continuous variables. Categorical variables were described with frequencies (%) and compared with chi-square test or Fisher's exact test. 5 missing BMI values were imputed using mean imputation in different gender.*

a *(Any AKI vs. NOT), Patients with any AKI (KDIGO stage1 or higher) compared with patients without AKI.*

b *(Severe AKI vs.NOT), Patients with severe AKI (KDIGO stage 2 and 3) compared with patients without AKI and patients with mild AKI (KDIGO stage1)*.

Patients who developed any AKI were with higher BMI and WBC. Additionally, they were more likely to experience longer operation time, CPB duration and more packed red blood cells (pRBC) transfusion. They also had more complicated postoperative course including longer ventilation time and ICU stays as well as higher rates of complications and 30-day mortality.

### Perioperative Kinetics of sMb and Correlation Between Different Biomarkers

**Figure 1:**
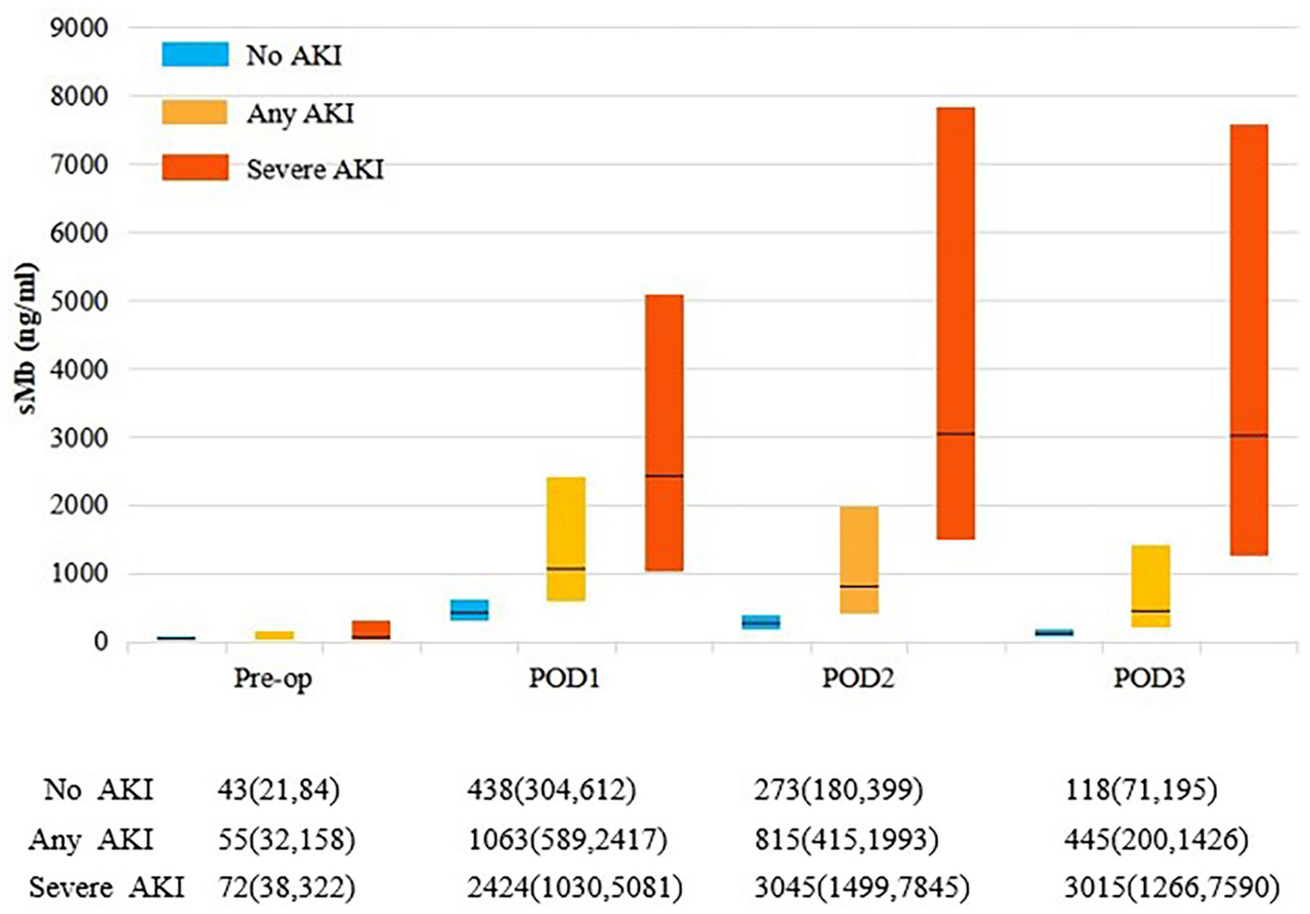
Perioperative sMb levels by AKI status. Each bar represents the interquartile range (25th percentile to 75th percentile) and the black line represents the median. Any AKI is KDIGO stage 1 or higher. Severe AKI is KDIGO stage 2 and 3.

Patients with any AKI had higher perioperative sMb levels than those without AKI. While patients with severe AKI had the highest levels in the three groups (Figure 1). The POD1 sMb level increased by 34-fold in severe AKI patients, 19-fold in any AKI patients, and 10-fold in patients without AKI when compared with preoperative sMb levels. The sMb level in patients without AKI reached peak on POD1. Then a rapid elimination was observed that the sMb level was decreased from peak concentration to 27% on POD 3. In patients with any AKI, sMb level reached peak on POD1 and eliminated to 42% of the peak on POD 3. In patients with severe AKI, the sMb level rose to peak on POD 2 and the concentration kept high on POD 3 (Figure 1). Patients with any AKI and severe AKI have a higher peak sMb level and a slower elimination rate. Meanwhile, the figure of daily changes in eGFR is shown as Supplementary Figure 3.

The POD1 values of all cardiac and renal biomarkers, including NT-proBNP, cTnI, CK-MB, CysC and sMb were significantly higher in patients with any AKI than in patients without AKI (Supplementary Table 1). Notably, we found weakly correlations between sMb and cardiac biomarkers. The renal biomarkers (AUC_CysC_) were also weakly correlated with cardiac biomarkers but strongly correlated with AUC_sMb_ (*r* = 0.639, *P* < 0.001) (Supplementary Table 2).

### Association of sMb With AKI

Both preoperative and postoperative logarithmically transformed sMb [Ln(sMb)] were associated with the different AKI severity in univariate models. The association persisted after adjusting for clinical covariates and changes of serum creatinine levels (ΔCr). Each unit increased in POD1 Ln(sMb) was independently associated with any AKI [adjusted OR 3.41 (95%CI, 1.67–6.98)] and severe AKI [adjusted OR 2.33 (95%CI, 1.49–3.65)]. Notably, the association of Ln(sMb) and patients with any AKI or severe AKI persisted after adjustment for logarithmically transformed NT-proBNP, cTnI, CK-MB, and CysC (Tables 2, 3). Thus, it indicated that sMb was capturing an unique aspect of the pathophysiology of AKI which differed from the above cardiac biomarkers and renal biomarker. And the adjusted ORs of Ln(sMb) for severe AKI on POD2 and POD3 are higher than the values on POD1.

**Table 2:** Association of Ln(sMb) with any AKI.

**Any AKI**	**Preoperative**		**Any AKI**	**POD1**		**POD2**		**POD3**	
	**OR (95% IC)**	***P*-value**		**OR (95% IC)**	***P*-value**	**OR (95% IC)**	***P*-value**	**OR (95% IC)**	***P*-value**
Ln(sMb)	1.45 (1.20, 1.76)	<0.001	Ln(sMb)	4.56 (3.16, 6.59)	<0.001	5.34 (3.62, 7.88)	<0.001	3.35 (2.51, 4.47)	<0.001
Ln(sMb)+Model	1.45 (1.16, 1.81)	0.001	Ln(sMb)+Model	4.53 (2.90, 7.07)	<0.001	5.65 (3.53, 9.05)	<0.001	3.15 (2.27, 4.36)	<0.001
Ln(sMb)+Model+ΔCr	NA	NA	Ln(sMb)+Model+ΔCr	3.41 (1.67, 6.98)	0.001	2.29 (1.24, 4.22)	0.008	2.02 (1.37, 2.98)	<0.001
Ln(sMb)+Model+ Ln(NT-proBNP)	1.45 (1.16, 1.81)	0.001	Ln(sMb)+Model+ΔCr +Ln(NT-proBNP)	2.88 (1.42, 5.85)	0.003	2.28 (1.22, 4.27)	0.010	1.97 (1.33, 2.93)	0.001
Ln(sMb)+Mode+ Ln(cTnI)	1.46 (1.16, 1.85)	0.001	Ln(sMb)+Model+ΔCr +Ln(cTnI)	2.90 (1.38, 6.09)	0.005	2.07 (1.09, 3.93)	0.026	1.90 (1.27, 2.83)	0.002
Ln(sMb)+Model+ Ln(CK-MB)	1.50 (1.11, 2.03)	0.008	Ln(sMb)+Model+ΔCr +Ln(CK-MB)	3.01 (1.43, 6.35)	0.004	2.20 (1.08, 4.52)	0.031	1.77 (1.13, 2.77)	0.013
Ln(sMb)+Model+ Ln(CysC)	1.37 (1.18, 1.71)	0.005	Ln(sMb)+Model+ΔCr +Ln(CysC)	2.36 (1.30, 5.77)	0.013	2.18 (1.18, 4.01)	0.013	1.82 (1.22, 2.71)	0.003

*Any AKI is KDIGO stage 1 or higher. Results are shown with continuous log-transformed sMb as the predictor variable. All odds ratios expressed as per unit increase in Ln(sMb). Preoperative Ln(sMb) was adjusted for Model^a^. Postoperative Ln(sMb) was adjusted for Model^b^. Model^a^, age, sex, BMI, hypertension, preoperative eGFR, preoperative WBC and preoperative lactate; Model^b^, age, sex, BMI, hypertension, preoperative eGFR and preoperative WBC, surgery duration, cardiopulmonary bypass duration, MHCA temperature and lactate*.

**Table 3:** Association of Ln(sMb) with severe AKI.

**Severe AKI**	**Preoperative**		**Severe AKI**	**POD1**		**POD2**		**POD3**	
	**OR (95% IC)**	***P*-value**		**OR (95% IC)**	***P*-value**	**OR (95% IC)**	***P*-value**	**OR (95% IC)**	***P*-value**
Ln(sMb)	1.51 (1.25, 1.82)	<0.001	Ln(sMb)	3.70 (2.68, 5.11)	<0.001	4.22 (3.08, 5.79)	<0.001	4.30 (3.13, 5.90)	<0.001
Ln(sMb)+Model	1.58 (1.26, 1.95)	<0.001	Ln(sMb)+Model	3.47 (2.27, 5.29)	<0.001	4.00 (2.72, 5.79)	<0.001	3.84 (2.70, 5.45)	<0.001
Ln(sMb)+Model +Δ Cr	NA	NA	Ln(sMb)+Model+ΔCr	2.33 (1.49, 3.65)	<0.001	2.56 (1.66, 3.94)	<0.001	3.00 (2.05, 4.38)	<0.001
Ln(sMb)+Model+ Ln(NT-proBNP)	1.56 (1.25, 1.95)	<0.001	Ln(sMb)+Model+ΔCr +Ln(NT-proBNP)	2.36 (1.47, 3.78)	<0.001	2.46 (1.60, 3.80)	<0.001	3.12 (2.12, 4.61)	<0.001
Ln(sMb)+Model+ Ln(cTnI)	1.54 (1.22, 1.95)	<0.001	Ln(sMb)+Model+ΔCr +Ln(cTnI)	2.15 (1.34, 3.44)	0.001	2.50 (1.57, 3.97)	<0.001	2.95 (1.96, 4.43)	<0.001
Ln(sMb) + Model + Ln(CK-MB)	1.68 (1.23, 2.29)	0.001	Ln(sMb)+Model+ΔCr +Ln(CK-MB)	2.28 (1.39, 3.75)	0.001	2.95 (1.74, 5.03)	<0.001	2.84 (1.78, 4.53)	<0.001
Ln(sMb)+Model+Ln(CysC)	1.48 (1.17, 1.86)	0.001	Ln(sMb)+Model+ΔCr +Ln(CysC)	2.15 (1.37, 3.39)	0.001	2.56 (1.66, 3.95)	<0.001	3.02 (2.05, 4.43)	<0.001

*Severe AKI is KDIGO stage 2 and 3. Results are shown with continuous log-transformed sMb as the predictor variable. All odds ratios expressed as per unit increase in Ln(sMb). Preoperative Ln(sMb) was adjusted for Model^a^. Postoperative Ln(sMb) was adjusted for Model^b^. Model^a^, age, sex, BMI, hypertension, preoperative eGFR, preoperative WBC and preoperative lactate; Model^b^, age, sex, BMI, hypertension, preoperative eGFR and preoperative WBC, surgery duration, cardiopulmonary bypass duration, MHCA temperature and lactate*.

### Association of sMb With Mortality

The postoperative 30-day mortality of the entire cohort was 11.8%. The mortality (35/75, 46.7%) of the patients with severe AKI is significantly higher than patients without AKI (1/130, 0.8%) (*P* < 0.001). The postoperative Ln(sMb) was associated with 30-day mortality after adjusted for logarithmically transformed individual renal biomarker and cardiac biomarkers. The relationship between preoperative Ln(sMb) and 30-day mortality was attenuated after adjusted for Ln(CK-MB) and Ln(CysC) (Supplementary Table 3). Multicollinearity is examined using the variance inflation factor (VIF) statistic and was found to be acceptable (Supplementary Table 5).

### Diagnostic Value of sMb for Clincial Outcomes

The area under the curve (AUC), event and non-event NRI and IDI for the different AKI severity and 30-day mortality of POD1 Ln(sMb) are presented in Table 4. POD1 Ln(sMb) improved the reclassification and discrimination of any AKI [ΔAUC = 0.08, (95% CI, 0.04–0.12), NRI = 0.74 (95% CI, 0.55–0.94), IDI = 0.13 (95%CI, 0.11–0.16)]. The same effect is observed for severe AKI and 30-day mortality.

**Table 4:** Prediction performance of the POD1 Ln(sMb).

	**Any AKI**	**Severe AKI**	**30-day mortality**
	**(95% IC)**	**(95% IC)**	**(95% IC)**
**AUC (95%IC)**			
Model^b^	0.75 (0.71, 0.79)	0.79 (0.75, 0.83)	0.74 (0.70, 0.78)
Model^b^+Ln(sMb)	0.83 (0.79, 0.87)	0.83 (0.81, 0.89)	0.79 (0.74, 0.82)
ΔAUC	0.08^**^ (0.04, 0.12)	0.08^*^ (0.04, 0.12)	0.05 (0.04, 0.12)
**Continuous NRI (95%)**			
All NRI	0.74^**^ (0.55, 0.94)	0.63^**^ (0.46, 0.81)	0.63^**^ (0.46, 0.81)
NRI+	0.29^**^ (0.21, 0.44)	0.31^**^ (0.15, 0.46)	0.34^**^ (0.03, 0.60)
NRI-	0.45^**^ (0.31, 0.59)	0.32^**^ (0.18, 0.47)	0.29^**^ (0.14, 0.43)
IDI	0.13^**^ (0.11, 0.16)	0.07^**^ (0.06, 0.09)	0.07^**^ (0.04, 0.09)

*Any AKI is KDIGO stage 1 or higher. Severe AKI is KDIGO stage 2 and 3.*

*Model^b^, age, sex, BMI, hypertension, preoperative eGFR and preoperative WBC, surgery duration, cardiopulmonary bypass duration, MHCA temperature and lactate.*

* *P-value < 0.05.*

** *P-value < 0.001*.

**Figure 2:**
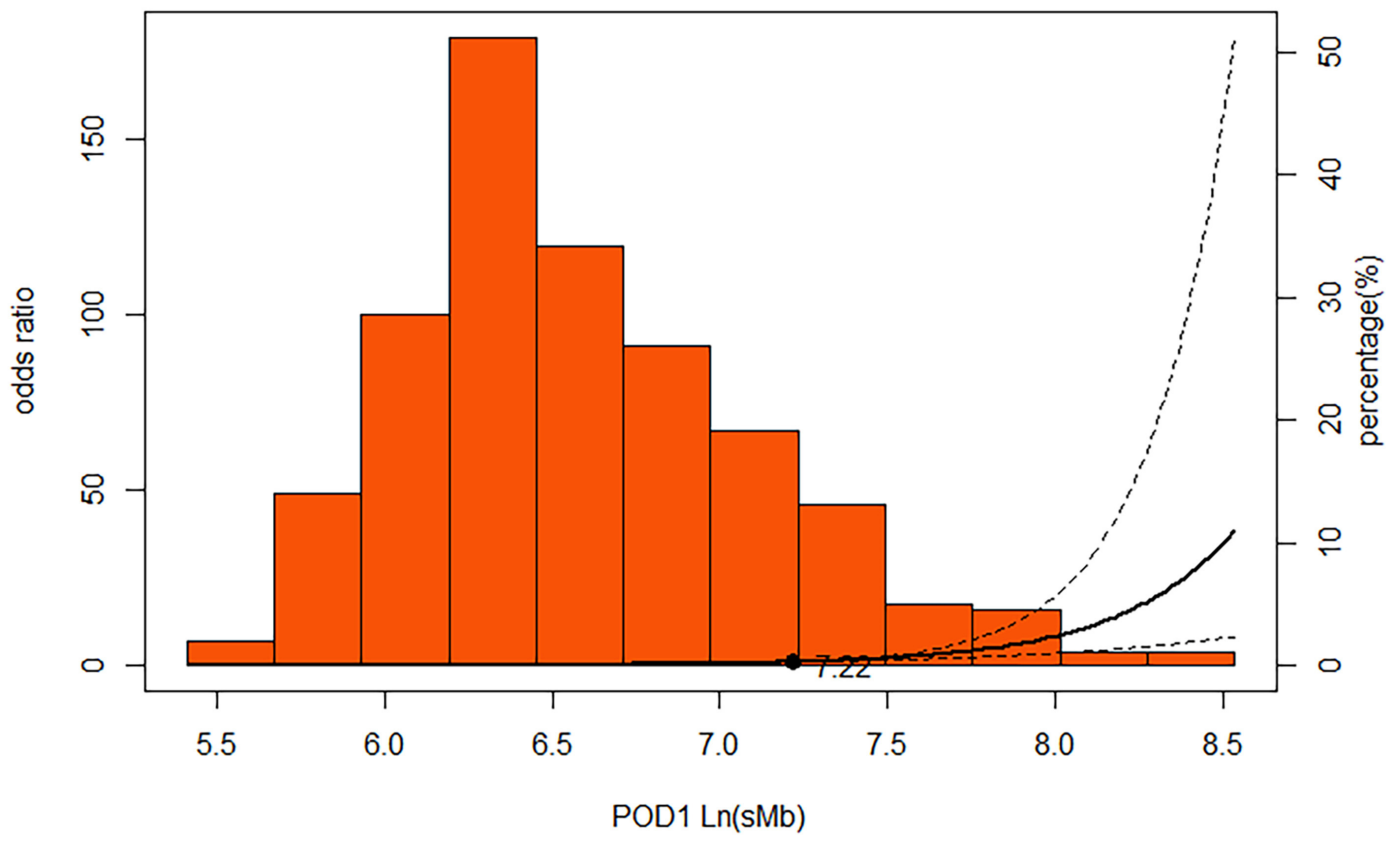
Multivariable-adjusted restricted cubic spline demonstrating that the adjusted association of POD1 Ln(sMb) with severe AKI was non-linear in persons underwent TAR with FET. Solid line represents ORs; dotted lines, 95% CIs. The extreme 5% of the data distribution was excluded to avoid implausible extrapolation from the extremes of the data. The spline function was adjusted for Model^b^. Model^b^: age, sex, BMI, hypertension, preoperative Egfr and preoperative WBC, surgery duration, cardiopulmonary bypass duration, the lowest rectal temperature and lactate.

ROC curve analysis showed that an POD1 Ln(sMb) concentration of >6.36 (corresponds to sMb = 580 ng/ml) predicted severe AKI with a sensitivity of 76.5% and a specificity of 72.3% [AUC = 0.796 (95% CI, 0.75–0.84)], and a value >7.22 (corresponds to sMb = 1380 ng/ml) predicted severe AKI with a sensitivity of 70.7% and a specificity of 79.9% [AUC = 0.809 (95% CI, 0.77–0.85)]. We found an inverse, but not J-shaped/U-shaped relationship between POD1 Ln(sMb) and severe AKI (*P* < 0.001) which suggest that the risk of severe AKI are positively correlated with the level of sMb (Figure 2). The diagnostic values of other other mentioned biomarkers are showed as Supplementary Table 6.

### Predictors of Postoperative sMb Concentration

Covariates with a *P*-value of <0.05 in univariate analysis were included into the multivariable analysis (*F* = 47.67, *P* < 0.001, adjusted *R*^2^ = 0.49) in terms of association with elevated POD1 Ln(sMb) level. Only weight, iliac artery involvement, preoperative eGFR, preoperative Ln(sMb), the lowest rectal temperature, surgery duration, pRBC transfusion and pH at ICU admission revealed significant influence on the POD1 Ln(sMb) level (Table 5). All other clinical factors mentioned in this article had no significant influence on postoperative sMb levels. The independent predictors of preoperative Ln(sMb) were gender, weight, preoperative WBC, preoperative eGFR and iliac artery involvement (Supplementary Table 4).

**Table 5:** Multiple linear regression of factors related to the POD1 Ln(sMb).

	***B***	**Std. error**	**β**	***P*-value**
(Constart)	9.663	3.308		0.004
Weight (kg)	0.007	0.003	0.081	0.039
Preoperative eGFR (ml/min/1.73m^2^)	−0.003	0.001	−0.103	0.012
Ln(Pre-op sMb) (ng/mL)	0.227	0.031	0.292	<0.001
Iliac artery involvement	0.260	0.076	0.129	0.001
MHCA temperature (°C)	0.247	0.047	0.203	<0.001
Surgery duration (min)	0.004	0.001	0.284	<0.001
pRBC transfusion (U)	0.023	0.004	0.216	<0.001
pH on ICU admission (mmol/L)	−1.694	0.396	−0.161	<0.001

*F = 47.665, P-value < 0.001, adjusted R^2^ = 0.485.*

*eGFR, estimated glomerular filtration rate; Pre-op, preoperative; sMb, serum myoglobin; MHCA, Moderate hypothermic circulatory arrest; pRBC, packed red blood cells; ICU, Intensive Care Unit*.

## Discussion

This study demonstrated that elevated preoperative and postoperative sMb were associated with AKI and 30-day mortality in TAAD patients underwent TAR with FET. After adjustment for clinical covariates, decline in filtration (via change in serum creatinine (ΔCr), cardiac biomarkers (via NT-proBNP, cTnI and CK-MB) and renal biomarker (via CysC), the statistical significance still existed. This argues that immediate myocardial injury and kindey injury can not fully explain this correlation. Moreover, only 3% patients developed oliguria in POD1, so it is unlikely that the immediate elevation of sMb levels postoperatively can be explained by impaired renal filtration. In addition, POD1 sMb improve the reclassification and discrimination of clinical model for the different AKI severity and 30-day mortality. The analysis by ROC revealed an adequate predictive accuracy of POD1 sMb to detect severe AKI for the optimal cut-off 1,380 ng/ml. The longer duration and higher level of sMb, the increased risk of severe AKI.

The pressure-related muscle injury causing rhabdomyolysis recognized in the patients undergoing prolonged surgeries, including the spinal, urological, bariatric and some cardiac surgeries (10–14). In these situations, skeletal muscle (mainly gluteal and back muscles) is more prone to hypoxic injury and vascular compromise because of its peripheral location and long-term compression (24). Consequently, many intracellular and often toxic components (e.g., CK, potassium and myoglobin among others) leak into the blood. Then, the myoglobin triggers the toxin causing renal dysfunction (25). Although, rhabdomyolysis is currently defined on the basis of CK which has a longer half-time. sMb has been proved to be more sensitive and specific than CK for predicting AKI (15, 26). In addition, compared with off-pump procedures, on-pump procedures were associated with significantly higher sMb peak concentrations following coronary artery bypass grafting (CABG) (13). This suggested that CPB might exacerbate rhabdomyolysis and increase sMb. It is notable that one study found that sMb is associated with renal dysfunction following thoracoabdominal aortic repairment (14). However, the study did not adjust for the occurrence of AKI as we did in our models. Hemodynamic management is not only related to rhabdomyolysis but plays a major role in development of AKI. Lactate was added in the Model for adjustment in regression to provide information on low perfusion. The correlation between lactate and sMb (*r* = 0.469, *P* < 0.001) is provided as Supplementary Figure 4.

This is the first study demonstrated that an elevated sMb level related to rhabdomyolysis is associated with AKI following TAR with FET. Current literatures provide several explanations for the association between sMb and AKI after TAR with FET. First, several studies have illustrated multiple pathologic factors, such as ischemia, compression and thrombosis result in rhabdomyolysis and release myoglobin (27). As a small-molecule protein, myoglobin can precipitate in the glomerular filtrate, particular in the acidic condition and hypovolemia, finally causing severe kidney damage. The mechanisms of myoglobin nephrotoxicity include: (1) formation of intratubular casts, (2) renal vasoconstriction, (3) heme-induced oxidative damage (27). Second, TAR with FET could only be accomplished with the aid of sophisticated CPB techniques, including variable arterial cannulation strategy, hypothermic circulatory arrest and selective cerebral perfusion. All these operations may worsen the hemodynamic instability and exacerbate skeletal muscle damage. It has been suggested that femoral arterial cannulation contribute to leg malperfusion (28, 29). Third, there is a paucity of data regarding AKI undergoing arch surgery. Current accepted mechanisms of cardiac surgery–associated acute kidney injury (CS-AKI) involve hemodynamic perturbation, inflammation and oxidative stress, CPB-related hemolysis etc. (17). Previous studies have demonstrated skeletal muscle damage is more prominent in aortic surgery than cardiac surgery which further suggested that rhabdomyolysis may be important supplement to the existing knowledge on the mechanism of AKI after aortic surgery (30). Moreover, study have shown that the highest concentration of serum myoglobin can be detected between 4 and 12 h following muscle injury which correspond to POD1 timepoint (30). As the results show, the preoperative and postoperative day 1–3 sMb were related with AKI which suggest rhabdomyolysis may contribute to AKI preoperatively, intraoperatively and postoperatively. Especially for those with persistently elevated sMb may stand for more serious rhabdomyolysis and greater harm on kidney.

Therefore, a sMb targeted strategy to lower the sMb level might be a potential treatment for preventing AKI following TAR with FET. Firstly, to reduce the risk of rhabdomyolysis in high-risk patients, such as obese subjects with the dissection iliac artery involvement, higher preoperative sMb and lower preoperative estimated glomerular filtration rate (eGFR), it might be useful to optimize cannulation strategy (e.g., sidearm cannulation), avoid hypotension, shorten operation time and MHCA duration (31–33). Secondly, alleviation vasoconstriction and reduction of sMb accumulation by administration of nitric oxide, avoidance of acidosis and volume depletion (25, 34). Most cases of limb ischemia can be solved after TAR with FET. For patients with sustained elevated myoglobin levels, blood purification with a cytokine adsorber might be a therapeutic option (35). Last but not least, longitudinal monitoring of sMb is essential to more closely monitor disease activity and therapeutic efficiency (26).

There are several limitations of the present study. First, due to the correlational nature of this study, a causal interpretation between the elevated sMb and postoperative AKI can not be drawn in this cohort of patients. But our results do suggest a possible causal relationship between rhabdomyolysis and AKI after TAR with FET. Second, despite all of these relationships persisted after adjustment for the decline in filtration and renal biomarker, the renal injury secondary to the renal malperfusion or free hemoglobin due to hemolysis could also work in the strong relationship between sMb and AKI (36). Although iliac involvement which related with sMb in this study always stands for extensive aortic dissection. We do not have detailed clinical data about the preoperative and postoperative status of the renal arteries.

## Conclusion

This is the first study to demonstrate that sMb could improve postoperative AKI risk classification for TAAD patients underwent TAR with FET. Rhabdomyolysis may be an important supplement to the existing knowledge on the mechanism of AKI. A sMb targeted strategy might be useful in the management of AKI after TAR with FET. However, future studies with more detailed clinical data are needed to further validate the relationship between sMb and AKI in this cohort of patients.

## Data Availability Statement

The original contributions presented in the study are included in the article/Supplementary Materials, further inquiries can be directed to the corresponding author.

## Ethics Statement

The studies involving human participants were reviewed and approved by the Ethics Committee of Xijing Hospital. Written informed consent for participation was not required for this study in accordance with the national legislation and the institutional requirements.

## Author Contributions

ZJ, WD, SY, and JL designed the clinical study. CY, PH, DW, and ZW collected clinical data and specimens. FF, CY, and PH analyzed the data. ZJ, SY, CY, and PH wrote the article. All authors read and approved the final manuscript.

## Funding

This work was supported by grants from Shaanxi Key Science and Technology Innovation Team Project (2019ZDLSF01-01-02, 2020SF-250) and the National Natural Science Foundation of China (51837011, 81870218, 82070503).

## Conflict of Interest

The authors declare that the research was conducted in the absence of any commercial or financial relationships that could be construed as a potential conflictof interest.

## Publisher's Note

All claims expressed in this article are solely those of the authors and do not necessarily represent those of their affiliated organizations, or those of the publisher, the editors and the reviewers. Any product that may be evaluated in this article, or claim that may be made by its manufacturer, is not guaranteed or endorsed by the publisher.

## Supplementary Material

Supplementary Figure 1 Flow chart of study exclusion and inclusion. TAAD, Type A aortic dissection; TAR, total arch replacement; FET, frozen elephant trunk; ESRD: AKI: acute kidney injury.

Supplementary Figure 2 Frozen elephant trunk (MicroPort Medical, Shanghai, China).

Supplementary Figure 3 Daily changes in eGFR.

Supplementary Figure 4 Correlation between lactate and Ln(sMb) on POD1.
