# Supplementary material for: Serum Myoglobin Is Associated With Postoperative Acute Kidney Injury in Stanford Type A Aortic Dissection
Source: Front Med (Lausanne). 2022 Feb 22;9:821418. doi: 10.3389/fmed.2022.821418 (PMC8902311; doi:10.3389/fmed.2022.821418)
Supplement: Supplementary file 1 [file Table_1.DOCX]

Table E1. Correlation coefficients of the AUC of sMb with other cardiac and renal biomarkers

|  | AUC_NT-proBNP_ | AUC_cTnI_ | AUC_CK-MB_ | AUC_CysC_ |
| --- | --- | --- | --- | --- |
| AUC_sMb_ | 0.339*^*^ | 0.591*^*^ | 0.587*^*^ | 0.639*^*^ |
| AUC_NT-proBNP_ | … | 0.369*^*^ | 0.315*^*^ | 0.336*^*^ |
| AUC_cTnI_ | … | … | 0.693*^*^ | 0.379*^*^ |
| AUC_CK-MB_ | … | … | … | 0.245*^*^ |

^**^*P*-value<0.001

The area under the curve (AUC_sMb_, AUC_NT-proBNP_, AUC_cTnI_, AUC_CK-MB_ and AUC_CysC_) was calculated for each patient using trapezoidal analysis with time as a baseline. The correlations were analyzed using Pearson’s correlation.
