# Supplementary material for: Serum Myoglobin Is Associated With Postoperative Acute Kidney Injury in Stanford Type A Aortic Dissection
Source: Front Med (Lausanne). 2022 Feb 22;9:821418. doi: 10.3389/fmed.2022.821418 (PMC8902311; doi:10.3389/fmed.2022.821418)
Supplement: Supplementary file 2 [file Table_2.DOCX]

Table E2. POD1 cardiac and renal biomarkers

| Biomarker | No AKI(n=130) | Any AKI(n=268) | Severe AKI(n=75) |
| --- | --- | --- | --- |
| sMb(ng/mL) | 438(304,612) | 1063(589,2417)^**^ | 2424(1030,5081)^##^ |
| NT-proBNP(pg/mL) | 1036(552,1593) | 1602(813,2900)^*^ | 2767(1346,3578)^#^ |
| cTnI(ng/mL) | 3.91(2.38) | 6.8(3.9,13.6)^**^ | 10.93(4.84,27.66)^##^ |
| CK-MB(ng/mL) | 20.5(13.9,28.8) | 28.9(17.6,48.1)^**^ | 38.2(19.3,72.2)^##^ |
| CysC(pg/mL) | 0.88(0.77,1.00) | 1.30(0.98,1.72)^**^ | 1.66(1.16,2.55)^##^ |

^*^P-value<0.05(Any AKI vs. NOT),^**^P-value<0.001(Any AKI vs. NOT)

^#^P-value<0.05(Severe AKI vs. NOT),^##^P-value<0.001(Severe AKI vs. NOT)

Any AKI is KDIGO stage 1 or higher. Severe AKI is KDIGO stage 2&3.

Any AKI vs. NOT: Patients with any AKI (KDIGO stage1 or higher) compared with patients without AKI.

Severe AKI vs.NOT: Patients with severe AKI (KDIGO stage 2&3) compared with patients without AKI and patients with mild AKI (KDIGO stage1).
