# Supplementary material for: Serum Myoglobin Is Associated With Postoperative Acute Kidney Injury in Stanford Type A Aortic Dissection
Source: Front Med (Lausanne). 2022 Feb 22;9:821418. doi: 10.3389/fmed.2022.821418 (PMC8902311; doi:10.3389/fmed.2022.821418)
Supplement: Supplementary file 3 [file Table_3.DOCX]

Table E4. Multiple linear regression of factors related to the Ln(Pre-op sMb)

|  | *B* | Std.Error | *β* | P-value |
| --- | --- | --- | --- | --- |
| (Constart) | 4.815 | .634 |  | .000 |
| Men | .644 | .160 | .201 | .000 |
| Age | .003 | .006 | .025 | .608 |
| Hypertension | .139 | .135 | .048 | .304 |
| Weight(kg) | -.015 | .006 | -.141 | .009 |
| WBC counts(10^9^/L) | .048 | .016 | .145 | .002 |
| Preoperative eGFR(ml/min/1.73m^2^) | -.013 | .002 | -.315 | .000 |
| Iliac artery involvement | .384 | .118 | .148 | .001 |

F=12.528, *P*-value<0.001, adjusted *R*^2^=0.222

Pre-op: preoperative; sMb: serum myoglobin; WBC: White blood cell; eGFR: estimated glomerular filtration rate
