# Supplementary material for: Serum Myoglobin Is Associated With Postoperative Acute Kidney Injury in Stanford Type A Aortic Dissection
Source: Front Med (Lausanne). 2022 Feb 22;9:821418. doi: 10.3389/fmed.2022.821418 (PMC8902311; doi:10.3389/fmed.2022.821418)
Supplement: Supplementary file 4 [file Table_4.DOCX]

Table E3. Association of Ln(sMb) with 30-day mortality

| 30-day mortality | Preoperative | | 30-day mortality | POD1 | | POD2 | | POD3 | |
| --- | --- | --- | --- | --- | --- | --- | --- | --- | --- |
|  | OR(95% IC) | P-value |  | OR(95% IC) | P-value | OR(95% IC) | P-value | OR(95% IC) | P-value |
| Ln(sMb) | 1.28(1.03,1.59) | 0.025 | Ln(sMb) | 2.04(1.52,2.74) | <0.001 | 2.41(1.87,3.10) | <0.001 | 2.26(1.82,2.81) | <0.001 |
| Ln(sMb)+Model | 1.33(1.04,1.70) | 0.021 | Ln(sMb)+Model | 2.01(1.33,3.04) | <0.001 | 2.45(1.74,3.45) | <0.001 | 2.26(1.71,2.99) | <0.001 |
| Ln(sMb)+Model+ΔCr | NA | NA | Ln(sMb)+Model+ΔCr | 1.88(1.17,3.02) | 0.009 | 2.23(1.51,3.30) | <0.001 | 2.22(1.60,3.07) | <0.001 |
| Ln(sMb)+Model+Ln(NT-proBNP) | 1.32(1.03,1.70) | 0.026 | Ln(sMb)+Model+ΔCr +Ln(NT-proBNP) | 1.81(1.11,2.96) | 0.018 | 2.13(1.43,3.16) | <0.001 | 2.18(1.57,3.02) | <0.001 |
| Ln(sMb)+Model+Ln(cTnI) | 1.33(1.03,1.72) | 0.032 | Ln(sMb)+Model+ΔCr +Ln(cTnI) | 1.85(1.11,3.06) | 0.017 | 2.06(1.35,3.15) | 0.001 | 2.23(1.57,3.18) | <0.001 |
| Ln(sMb)+Model+Ln(CK-MB) | 1.26(0.90,1.76) | 0.187 | Ln(sMb)+Model+ΔCr +Ln(CK-MB) | 1.77(1.05,2.99) | 0.033 | 2.72(1.67,4.44) | <0.001 | 2.80(1.79,4.38) | <0.001 |
| Ln(sMb)+Model+Ln(CysC) | 1.24(0.96,1.61) | 0.101 | Ln(sMb)+Model+ΔCr +Ln(CysC) | 1.78(1.10,2.89) | 0.020 | 2.23(1.51,3.30) | <0.001 | 2.23(1.60,3.10) | <0.001 |

Results are shown with continuous log-transformed sMb as the predictor variable. All odds ratios expressed as per unit increase in Ln(sMb).

Preoperative Ln(sMb) was adjusted for Model^a^. Postoperative Ln(sMb) was adjusted for Model^b^. Model^a^: age, sex, BMI, hypertension, preoperative eGFR, preoperative WBC and preoperative lactate. Model^b^: age, sex, BMI, hypertension, preoperative eGFR and preoperative WBC, surgery duration, cardiopulmonary bypass duration, MHCA temperature and lactate.
