# Supplementary material for: Serum Myoglobin Is Associated With Postoperative Acute Kidney Injury in Stanford Type A Aortic Dissection
Source: Front Med (Lausanne). 2022 Feb 22;9:821418. doi: 10.3389/fmed.2022.821418 (PMC8902311; doi:10.3389/fmed.2022.821418)
Supplement: Supplementary file 5 [file Table_5.DOCX]

1.Collinearity Statistics of Lnpre-sMb+ Model^a^ with any AKI Regression Analysis

|  | Collinearity Statistics | |
| --- | --- | --- |
|  | Tolerance | VIF |
| Male | 0.899 | 1.113 |
| Age | 0.843 | 1.186 |
| BMI | 0.859 | 1.164 |
| Hypertension | 0.904 | 1.106 |
| Pre-eGFR | 0.778 | 1.286 |
| Pre-WBC | 0.821 | 1.218 |
| Pre-Lac | 0.88 | 1.137 |
| Lnpre-sMb | 0.792 | 1.263 |

2.Collinearity Statistics of Lnpre-sMb+Model^a^+Lnpre-BNP with any AKI Regression Analysis

|  | Collinearity Statistics | |
| --- | --- | --- |
|  | Tolerance | VIF |
| Male | 0.897 | 1.115 |
| Age | 0.842 | 1.188 |
| BMI | 0.851 | 1.176 |
| Hypertension | 0.902 | 1.108 |
| Pre-eGFR | 0.761 | 1.314 |
| Pre-WBC | 0.818 | 1.223 |
| Pre-Lac | 0.88 | 1.137 |
| Lnpre-sMb | 0.783 | 1.277 |
| Lnpre-BNP | 0.929 | 1.076 |

3.Collinearity Statistics of Lnpre-sMb+Model^a^+Lnpre-CK_MB with any AKI Regression Analysis

|  | Collinearity Statistics | |
| --- | --- | --- |
|  | Tolerance | VIF |
| Male | 0.897 | 1.115 |
| Age | 0.841 | 1.19 |
| BMI | 0.857 | 1.167 |
| Hypertension | 0.896 | 1.116 |
| Pre-eGFR | 0.777 | 1.286 |
| Pre-WBC | 0.807 | 1.24 |
| Pre-Lac | 0.878 | 1.139 |
| Lnpre-sMb | 0.419 | 2.384 |
| Lnpre-CK_MB | 0.457 | 2.187 |

4.Collinearity Statistics of Lnpre-sMb+Model^a^+Lnpre-CysC with any AKI Regression Analysis

|  | Collinearity Statistics | |
| --- | --- | --- |
|  | Tolerance | VIF |
| Male | 0.867 | 1.153 |
| Age | 0.843 | 1.186 |
| BMI | 0.859 | 1.164 |
| Hypertension | 0.904 | 1.107 |
| Pre-eGFR | 0.544 | 1.84 |
| Pre-WBC | 0.815 | 1.227 |
| Pre-Lac | 0.874 | 1.144 |
| Lnpre-sMb | 0.758 | 1.318 |
| Lnpre-CysC | 0.552 | 1.812 |

5.Collinearity Statistics of Lnpre-sMb+Model^a^ with Severe AKI Regression Analysis

|  | Collinearity Statistics | |
| --- | --- | --- |
|  | Tolerance | VIF |
| Male | 0.899 | 1.113 |
| Age | 0.843 | 1.186 |
| BMI | 0.859 | 1.164 |
| Hypertension | 0.904 | 1.106 |
| Pre-eGFR | 0.778 | 1.286 |
| Pre-WBC | 0.821 | 1.218 |
| Pre-Lac | 0.88 | 1.137 |
| Lnpre-sMb | 0.792 | 1.263 |

6.Collinearity Statistics of Lnpre-sMb+Model^a^+Lnpre-BNP with Severe AKI Regression Analysis

|  | Collinearity Statistics | |
| --- | --- | --- |
|  | Tolerance | VIF |
| Male | 0.897 | 1.115 |
| Age | 0.842 | 1.188 |
| BMI | 0.851 | 1.176 |
| Hypertension | 0.902 | 1.108 |
| Pre-eGFR | 0.761 | 1.314 |
| Pre-WBC | 0.818 | 1.223 |
| Pre-Lac | 0.88 | 1.137 |
| Lnpre-sMb | 0.783 | 1.277 |
| Lnpre-BNP | 0.929 | 1.076 |

7.Collinearity Statistics of Lnpre-sMb+Model^a^+Lnpre-cTnI with Severe AKI Regression Analysis

|  | Collinearity Statistics | |
| --- | --- | --- |
|  | Tolerance | VIF |
| Male | 0.898 | 1.113 |
| Age | 0.842 | 1.187 |
| BMI | 0.857 | 1.166 |
| Hypertension | 0.903 | 1.108 |
| Pre-eGFR | 0.772 | 1.296 |
| Pre-WBC | 0.818 | 1.222 |
| Pre-Lac | 0.876 | 1.142 |
| Lnpre-sMb | 0.708 | 1.412 |
| Lnpre-cTnI | 0.813 | 1.23 |

8.Collinearity Statistics of Lnpre-sMb+Model^a^+ Lnpre-CK_MB with Severe AKI Regression Analysis

|  | Collinearity Statistics | |
| --- | --- | --- |
|  | Tolerance | VIF |
| Male | 0.897 | 1.115 |
| Age | 0.841 | 1.19 |
| BMI | 0.857 | 1.167 |
| Hypertension | 0.896 | 1.116 |
| Pre-eGFR | 0.777 | 1.286 |
| Pre-WBC | 0.807 | 1.24 |
| Pre-Lac | 0.878 | 1.139 |
| Lnpre-sMb | 0.419 | 2.384 |
| Lnpre-CK_MB | 0.457 | 2.187 |

9.Collinearity Statistics of Lnpre-sMb+Model^a^+Lnpre-CysC with Severe AKI Regression Analysis

|  | Collinearity Statistics | |
| --- | --- | --- |
|  | Tolerance | VIF |
| Male | 0.867 | 1.153 |
| Age | 0.843 | 1.186 |
| BMI | 0.859 | 1.164 |
| Hypertension | 0.904 | 1.107 |
| Pre-eGFR | 0.544 | 1.84 |
| Pre-WBC | 0.815 | 1.227 |
| Pre-Lac | 0.874 | 1.144 |
| Lnpre-sMb | 0.758 | 1.318 |
| Lnpre-CysC | 0.552 | 1.812 |

10.Collinearity Statistics of Lnpre-sMb+Model^a^ with 30-day Mortality Regression Analysis

|  | Collinearity Statistics | |
| --- | --- | --- |
|  | Tolerance | VIF |
| Male | 0.899 | 1.113 |
| Age | 0.843 | 1.186 |
| BMI | 0.859 | 1.164 |
| Hypertension | 0.904 | 1.106 |
| Pre-eGFR | 0.778 | 1.286 |
| Pre-WBC | 0.821 | 1.218 |
| Pre-Lac | 0.88 | 1.137 |
| Lnpre-sMb | 0.792 | 1.263 |

11.Collinearity Statistics of Lnpre-sMb+Model^a^+Lnpre-BNP with 30-day Mortality Regression Analysis

|  | Collinearity Statistics | |
| --- | --- | --- |
|  | Tolerance | VIF |
| Male | 0.897 | 1.115 |
| Age | 0.842 | 1.188 |
| BMI | 0.851 | 1.176 |
| Hypertension | 0.902 | 1.108 |
| Pre-eGFR | 0.761 | 1.314 |
| Pre-WBC | 0.818 | 1.223 |
| Pre-Lac | 0.88 | 1.137 |
| Lnpre-sMb | 0.783 | 1.277 |
| Lnpre-BNP | 0.929 | 1.076 |

12.Collinearity Statistics of Lnpre-sMb+Model^a^+Lnpre-cTnI with 30-day Mortality Regression Analysis

|  | Collinearity Statistics | |
| --- | --- | --- |
|  | Tolerance | VIF |
| Male | 0.898 | 1.113 |
| Age | 0.842 | 1.187 |
| BMI | 0.857 | 1.166 |
| Hypertension | 0.903 | 1.108 |
| Pre-eGFR | 0.772 | 1.296 |
| Pre-WBC | 0.818 | 1.222 |
| Pre-Lac | 0.876 | 1.142 |
| Lnpre-sMb | 0.708 | 1.412 |
| Lnpre-cTnI | 0.813 | 1.23 |

13.Collinearity Statistics of Lnpre-sMb+Model^a^+Lnpre-CK_MB with 30-day Mortality Regression Analysis

|  | Collinearity Statistics | |
| --- | --- | --- |
|  | Tolerance | VIF |
| Male | 0.897 | 1.115 |
| Age | 0.841 | 1.19 |
| BMI | 0.857 | 1.167 |
| Hypertension | 0.896 | 1.116 |
| Pre-eGFR | 0.777 | 1.286 |
| Pre-WBC | 0.807 | 1.24 |
| Pre-Lac | 0.878 | 1.139 |
| Lnpre-sMb | 0.419 | 2.384 |
| Lnpre-CK_MB | 0.457 | 2.187 |

14.Collinearity Statistics of Lnpre-sMb+Model^a^+Lnpre-CysC with 30-day Mortality Regression Analysis

|  | Collinearity Statistics | |
| --- | --- | --- |
|  | Tolerance | VIF |
| Male | 0.867 | 1.153 |
| Age | 0.843 | 1.186 |
| BMI | 0.859 | 1.164 |
| Hypertension | 0.904 | 1.107 |
| Pre-eGFR | 0.544 | 1.84 |
| Pre-WBC | 0.815 | 1.227 |
| Pre-Lac | 0.874 | 1.144 |
| Lnpre-sMb | 0.758 | 1.318 |
| Lnpre-CysC | 0.552 | 1.812 |

15.Collinearity Statistics of LnPOD1sMb+Model^b^ with any AKI Regression Analysis

|  | Collinearity Statistics | |
| --- | --- | --- |
|  | Tolerance | VIF |
| Male | 0.893 | 1.12 |
| Age | 0.84 | 1.191 |
| BMI | 0.818 | 1.223 |
| Hypertension | 0.909 | 1.101 |
| Pre-eGFR | 0.791 | 1.265 |
| Pre-WBC | 0.835 | 1.197 |
| Surgery duration | 0.382 | 2.618 |
| CPB duration | 0.399 | 2.505 |
| MHCA temperature | 0.789 | 1.267 |
| POD1Lac | 0.812 | 1.231 |
| LnPOD1sMb | 0.616 | 1.622 |

16.Collinearity Statistics of LnPOD1sMb+Model^b^+ΔCr with any AKI Regression Analysis

|  | Collinearity Statistics | |
| --- | --- | --- |
|  | Tolerance | VIF |
| Male | 0.892 | 1.121 |
| Age | 0.839 | 1.191 |
| BMI | 0.809 | 1.236 |
| Hypertension | 0.906 | 1.103 |
| Pre-eGFR | 0.78 | 1.283 |
| Pre-WBC | 0.833 | 1.2 |
| Surgery duration | 0.381 | 2.623 |
| CPB duration | 0.399 | 2.508 |
| MHCA temperature | 0.789 | 1.267 |
| POD1Lac | 0.772 | 1.296 |
| LnPOD1sMb | 0.475 | 2.104 |
| ΔCr | 0.574 | 1.742 |

17.Collinearity Statistics of LnPOD1sMb+Model^b^+ΔCr+LnPOD1BNP with any AKI Regression Analysis

|  | Collinearity Statistics | |
| --- | --- | --- |
|  | Tolerance | VIF |
| Male | 0.863 | 1.159 |
| Age | 0.839 | 1.191 |
| BMI | 0.797 | 1.255 |
| Hypertension | 0.899 | 1.112 |
| Pre-eGFR | 0.777 | 1.288 |
| Pre-WBC | 0.833 | 1.2 |
| Surgery duration | 0.375 | 2.666 |
| CPB duration | 0.386 | 2.588 |
| MHCA temperature | 0.763 | 1.311 |
| POD1Lac | 0.721 | 1.387 |
| LnPOD1sMb | 0.465 | 2.149 |
| ΔCr | 0.569 | 1.758 |
| LnPOD1BNP | 0.705 | 1.419 |

18.Collinearity Statistics of LnPOD1sMb+Model^b^+ΔCr+LnPOD1cTnI with any AKI Regression Analysis

|  | Collinearity Statistics | |
| --- | --- | --- |
|  | Tolerance | VIF |
| Male | 0.887 | 1.128 |
| Age | 0.838 | 1.193 |
| BMI | 0.807 | 1.24 |
| Hypertension | 0.9 | 1.111 |
| Pre-eGFR | 0.777 | 1.288 |
| Pre-WBC | 0.811 | 1.233 |
| Surgery duration | 0.381 | 2.626 |
| CPB duration | 0.395 | 2.531 |
| MHCA temperature | 0.788 | 1.269 |
| POD1Lac | 0.744 | 1.344 |
| LnPOD1sMb | 0.423 | 2.363 |
| ΔCr | 0.571 | 1.75 |
| LnPOD1cTnI | 0.615 | 1.626 |

19.Collinearity Statistics of LnPOD1sMb+Model^b^+ΔCr+LnPOD1CK_MB with any AKI Regression Analysis

|  | Collinearity Statistics | |
| --- | --- | --- |
|  | Tolerance | VIF |
| Male | 0.891 | 1.122 |
| Age | 0.838 | 1.193 |
| BMI | 0.806 | 1.24 |
| Hypertension | 0.888 | 1.126 |
| Pre-eGFR | 0.77 | 1.299 |
| Pre-WBC | 0.828 | 1.208 |
| Surgery duration | 0.379 | 2.637 |
| CPB duration | 0.394 | 2.537 |
| MHCA temperature | 0.785 | 1.273 |
| POD1Lac | 0.752 | 1.329 |
| LnPOD1sMb | 0.396 | 2.527 |
| ΔCr | 0.57 | 1.753 |
| LnPOD1CK_MB | 0.675 | 1.481 |

20.Collinearity Statistics of LnPOD1sMb+Model^b^+ΔCr+LnPOD1CysC with any AKI Regression Analysis

|  | Collinearity Statistics | |
| --- | --- | --- |
|  | Tolerance | VIF |
| Male | 0.876 | 1.142 |
| Age | 0.838 | 1.193 |
| BMI | 0.803 | 1.245 |
| Hypertension | 0.88 | 1.136 |
| Pre-eGFR | 0.616 | 1.623 |
| Pre-WBC | 0.831 | 1.204 |
| Surgery duration | 0.379 | 2.635 |
| CPB duration | 0.398 | 2.512 |
| MHCA temperature | 0.788 | 1.269 |
| POD1Lac | 0.771 | 1.296 |
| LnPOD1sMb | 0.451 | 2.218 |
| ΔCr | 0.377 | 2.649 |
| LnPOD1CysC | 0.332 | 3.009 |

21.Collinearity Statistics of LnPOD1sMb+Model^b^ with Severe AKI Regression Analysis

|  | Collinearity Statistics | |
| --- | --- | --- |
|  | Tolerance | VIF |
| Male | 0.893 | 1.12 |
| Age | 0.84 | 1.191 |
| BMI | 0.818 | 1.223 |
| Hypertension | 0.909 | 1.101 |
| Pre-eGFR | 0.791 | 1.265 |
| Pre-WBC | 0.835 | 1.197 |
| Surgery duration | 0.382 | 2.618 |
| CPB duration | 0.399 | 2.505 |
| MHCA temperature | 0.789 | 1.267 |
| POD1Lac | 0.812 | 1.231 |
| LnPOD1sMb | 0.616 | 1.622 |

22.Collinearity Statistics of LnPOD1sMb+Model^b^+ΔCr with Severe AKI Regression Analysis

|  | Collinearity Statistics | |
| --- | --- | --- |
|  | Tolerance | VIF |
| Male | 0.892 | 1.121 |
| Age | 0.839 | 1.191 |
| BMI | 0.809 | 1.236 |
| Hypertension | 0.906 | 1.103 |
| Pre-eGFR | 0.78 | 1.283 |
| Pre-WBC | 0.833 | 1.2 |
| Surgery duration | 0.381 | 2.623 |
| CPB duration | 0.399 | 2.508 |
| MHCA temperature | 0.789 | 1.267 |
| POD1Lac | 0.772 | 1.296 |
| LnPOD1sMb | 0.475 | 2.104 |
| ΔCr | 0.574 | 1.742 |

23.Collinearity Statistics of LnPOD1sMb+Model^b^+ΔCr+LnPOD1BNP with Severe AKI Regression Analysis

|  | Collinearity Statistics | |
| --- | --- | --- |
|  | Tolerance | VIF |
| Male | 0.863 | 1.159 |
| Age | 0.839 | 1.191 |
| BMI | 0.797 | 1.255 |
| Hypertension | 0.899 | 1.112 |
| Pre-eGFR | 0.777 | 1.288 |
| Pre-WBC | 0.833 | 1.2 |
| Surgery duration | 0.375 | 2.666 |
| CPB duration | 0.386 | 2.588 |
| MHCA temperature | 0.763 | 1.311 |
| POD1Lac | 0.721 | 1.387 |
| LnPOD1sMb | 0.465 | 2.149 |
| ΔCr | 0.569 | 1.758 |
| LnPOD1BNP | 0.705 | 1.419 |

24.Collinearity Statistics of LnPOD1sMb+Model^b^+ΔCr+LnPOD1cTnI with Severe AKI Regression Analysis

|  | Collinearity Statistics | |
| --- | --- | --- |
|  | Tolerance | VIF |
| Male | 0.887 | 1.128 |
| Age | 0.838 | 1.193 |
| BMI | 0.807 | 1.24 |
| Hypertension | 0.9 | 1.111 |
| Pre-eGFR | 0.777 | 1.288 |
| Pre-WBC | 0.811 | 1.233 |
| Surgery duration | 0.381 | 2.626 |
| CPB duration | 0.395 | 2.531 |
| MHCA temperature | 0.788 | 1.269 |
| POD1Lac | 0.744 | 1.344 |
| LnPOD1sMb | 0.423 | 2.363 |
| ΔCr | 0.571 | 1.75 |
| LnPOD1cTnI | 0.615 | 1.626 |

25.Collinearity Statistics of LnPOD1sMb+Model^b^+ΔCr+LnPOD1CK_MB with Severe AKI Regression Analysis

|  | Collinearity Statistics | |
| --- | --- | --- |
|  | Tolerance | VIF |
| Male | 0.891 | 1.122 |
| Age | 0.838 | 1.193 |
| BMI | 0.806 | 1.24 |
| Hypertension | 0.888 | 1.126 |
| Pre-eGFR | 0.77 | 1.299 |
| Pre-WBC | 0.828 | 1.208 |
| Surgery duration | 0.379 | 2.637 |
| CPB duration | 0.394 | 2.537 |
| MHCA temperature | 0.785 | 1.273 |
| POD1Lac | 0.752 | 1.329 |
| LnPOD1sMb | 0.396 | 2.527 |
| ΔCr | 0.57 | 1.753 |
| LnPOD1CK_MB | 0.675 | 1.481 |

26.Collinearity Statistics of LnPOD1sMb+Model^b^+ΔCr+LnPOD1CysC with Severe AKI Regression Analysis

|  | Collinearity Statistics | |
| --- | --- | --- |
|  | Tolerance | VIF |
| Male | 0.876 | 1.142 |
| Age | 0.838 | 1.193 |
| BMI | 0.803 | 1.245 |
| Hypertension | 0.88 | 1.136 |
| Pre-eGFR | 0.616 | 1.623 |
| Pre-WBC | 0.831 | 1.204 |
| Surgery duration | 0.379 | 2.635 |
| CPB duration | 0.398 | 2.512 |
| MHCA temperature | 0.788 | 1.269 |
| POD1Lac | 0.771 | 1.296 |
| LnPOD1sMb | 0.451 | 2.218 |
| ΔCr | 0.377 | 2.649 |
| LnPOD1CysC | 0.332 | 3.009 |

27.Collinearity Statistics of LnPOD1sMb+Model^b^ with 30-day Mortality Regression Analysis

|  | Collinearity Statistics | |
| --- | --- | --- |
|  | Tolerance | VIF |
| Male | 0.893 | 1.12 |
| Age | 0.84 | 1.191 |
| BMI | 0.818 | 1.223 |
| Hypertension | 0.909 | 1.101 |
| Pre-eGFR | 0.791 | 1.265 |
| Pre-WBC | 0.835 | 1.197 |
| Surgery duration | 0.382 | 2.618 |
| CPB duration | 0.399 | 2.505 |
| MHCA temperature | 0.789 | 1.267 |
| POD1Lac | 0.812 | 1.231 |
| LnPOD1sMb | 0.616 | 1.622 |

28.Collinearity Statistics of LnPOD1sMb+Model^b^+ΔCr with 30-day Mortality Regression Analysis

|  | Collinearity Statistics | |
| --- | --- | --- |
|  | Tolerance | VIF |
| Male | 0.892 | 1.121 |
| Age | 0.839 | 1.191 |
| BMI | 0.809 | 1.236 |
| Hypertension | 0.906 | 1.103 |
| Pre-eGFR | 0.78 | 1.283 |
| Pre-WBC | 0.833 | 1.2 |
| Surgery duration | 0.381 | 2.623 |
| CPB duration | 0.399 | 2.508 |
| MHCA temperature | 0.789 | 1.267 |
| POD1Lac | 0.772 | 1.296 |
| LnPOD1sMb | 0.475 | 2.104 |
| ΔCr | 0.574 | 1.742 |

29.Collinearity Statistics of LnPOD1sMb+Model^b^+ΔCr+LnPOD1BNP with 30-day Mortality Regression Analysis

|  | Collinearity Statistics | |
| --- | --- | --- |
|  | Tolerance | VIF |
| Male | 0.863 | 1.159 |
| Age | 0.839 | 1.191 |
| BMI | 0.797 | 1.255 |
| Hypertension | 0.899 | 1.112 |
| Pre-eGFR | 0.777 | 1.288 |
| Pre-WBC | 0.833 | 1.2 |
| Surgery duration | 0.375 | 2.666 |
| CPB duration | 0.386 | 2.588 |
| MHCA temperature | 0.763 | 1.311 |
| POD1Lac | 0.721 | 1.387 |
| LnPOD1sMb | 0.465 | 2.149 |
| ΔCr | 0.569 | 1.758 |
| LnPOD1BNP | 0.705 | 1.419 |

30.Collinearity Statistics of LnPOD1sMb+Model^b^+ΔCr+LnPOD1cTnI with 30-day Mortality Regression Analysis

|  | Collinearity Statistics | |
| --- | --- | --- |
|  | Tolerance | VIF |
| Male | 0.887 | 1.128 |
| Age | 0.838 | 1.193 |
| BMI | 0.807 | 1.24 |
| Hypertension | 0.9 | 1.111 |
| Pre-eGFR | 0.777 | 1.288 |
| Pre-WBC | 0.811 | 1.233 |
| Surgery duration | 0.381 | 2.626 |
| CPB duration | 0.395 | 2.531 |
| MHCA temperature | 0.788 | 1.269 |
| POD1Lac | 0.744 | 1.344 |
| LnPOD1sMb | 0.423 | 2.363 |
| ΔCr | 0.571 | 1.75 |
| LnPOD1cTnI | 0.615 | 1.626 |

31.Collinearity Statistics of LnPOD1sMb+Model^b^+ΔCr+LnPOD1CK_MB with 30-day Mortality Regression Analysis

|  | Collinearity Statistics | |
| --- | --- | --- |
|  | Tolerance | VIF |
| Male | 0.891 | 1.122 |
| Age | 0.838 | 1.193 |
| BMI | 0.806 | 1.24 |
| Hypertension | 0.888 | 1.126 |
| Pre-eGFR | 0.77 | 1.299 |
| Pre-WBC | 0.828 | 1.208 |
| Surgery duration | 0.379 | 2.637 |
| CPB duration | 0.394 | 2.537 |
| MHCA temperature | 0.785 | 1.273 |
| POD1Lac | 0.752 | 1.329 |
| LnPOD1sMb | 0.396 | 2.527 |
| ΔCr | 0.57 | 1.753 |
| LnPOD1CK_MB | 0.675 | 1.481 |

32.Collinearity Statistics of LnPOD1sMb+Model^b^+ΔCr+LnPOD1CysC with 30-day Mortality Regression Analysis

|  | Collinearity Statistics | |
| --- | --- | --- |
|  | Tolerance | VIF |
| Male | 0.876 | 1.142 |
| Age | 0.838 | 1.193 |
| BMI | 0.803 | 1.245 |
| Hypertension | 0.88 | 1.136 |
| Pre-eGFR | 0.616 | 1.623 |
| Pre-WBC | 0.831 | 1.204 |
| Surgery duration | 0.379 | 2.635 |
| CPB duration | 0.398 | 2.512 |
| MHCA temperature | 0.788 | 1.269 |
| POD1Lac | 0.771 | 1.296 |
| LnPOD1sMb | 0.451 | 2.218 |
| ΔCr | 0.377 | 2.649 |
| LnPOD1CysC | 0.332 | 3.009 |

33.Collinearity Statistics of LnPOD2sMb+Model^b^ with any AKI Regression Analysis

|  | Collinearity Statistics | |
| --- | --- | --- |
|  | Tolerance | VIF |
| Male | 0.905 | 1.105 |
| Age | 0.845 | 1.184 |
| BMI | 0.812 | 1.232 |
| Hypertension | 0.896 | 1.116 |
| Pre-eGFR | 0.825 | 1.212 |
| Pre-WBC | 0.852 | 1.174 |
| Surgery duration | 0.389 | 2.57 |
| CPB duration | 0.396 | 2.527 |
| MHCA temperature | 0.821 | 1.218 |
| POD2Lac | 0.842 | 1.188 |
| LnPOD2sMb | 0.62 | 1.613 |

34.Collinearity Statistics of LnPOD2sMb+Model^b^+ΔCr with any AKI Regression Analysis

|  | Collinearity Statistics | |
| --- | --- | --- |
|  | Tolerance | VIF |
| Male | 0.905 | 1.105 |
| Age | 0.844 | 1.185 |
| BMI | 0.799 | 1.252 |
| Hypertension | 0.889 | 1.125 |
| Pre-eGFR | 0.798 | 1.253 |
| Pre-WBC | 0.852 | 1.174 |
| Surgery duration | 0.389 | 2.57 |
| CPB duration | 0.396 | 2.528 |
| MHCA temperature | 0.82 | 1.219 |
| POD2Lac | 0.829 | 1.206 |
| LnPOD2sMb | 0.403 | 2.484 |
| ΔCr | 0.491 | 2.036 |

35.Collinearity Statistics of LnPOD2sMb+Model^b^+ΔCr+LnPOD2BNP with any AKI Regression Analysis

|  | Collinearity Statistics | |
| --- | --- | --- |
|  | Tolerance | VIF |
| Male | 0.891 | 1.122 |
| Age | 0.842 | 1.187 |
| BMI | 0.785 | 1.274 |
| Hypertension | 0.878 | 1.139 |
| Pre-eGFR | 0.797 | 1.254 |
| Pre-WBC | 0.85 | 1.176 |
| Surgery duration | 0.387 | 2.581 |
| CPB duration | 0.39 | 2.564 |
| MHCA temperature | 0.803 | 1.245 |
| POD2Lac | 0.823 | 1.215 |
| LnPOD2sMb | 0.392 | 2.553 |
| ΔCr | 0.489 | 2.043 |
| LnPOD2BNP | 0.836 | 1.196 |

36.Collinearity Statistics of LnPOD2sMb+Model^b^+ΔCr+LnPOD2cTnI with any AKI Regression Analysis

|  | Collinearity Statistics | |
| --- | --- | --- |
|  | Tolerance | VIF |
| Male | 0.898 | 1.114 |
| Age | 0.844 | 1.185 |
| BMI | 0.792 | 1.263 |
| Hypertension | 0.882 | 1.133 |
| Pre-eGFR | 0.798 | 1.253 |
| Pre-WBC | 0.83 | 1.205 |
| Surgery duration | 0.389 | 2.57 |
| CPB duration | 0.391 | 2.56 |
| MHCA temperature | 0.82 | 1.219 |
| POD2Lac | 0.821 | 1.217 |
| LnPOD2sMb | 0.335 | 2.982 |
| ΔCr | 0.488 | 2.051 |
| LnPOD2cTnI | 0.508 | 1.968 |

37.Collinearity Statistics of LnPOD2sMb+Model^b^+ΔCr+LnPOD2CK_MB with any AKI Regression Analysis

|  | Collinearity Statistics | |
| --- | --- | --- |
|  | Tolerance | VIF |
| Male | 0.901 | 1.11 |
| Age | 0.844 | 1.185 |
| BMI | 0.746 | 1.341 |
| Hypertension | 0.886 | 1.129 |
| Pre-eGFR | 0.79 | 1.267 |
| Pre-WBC | 0.849 | 1.178 |
| Surgery duration | 0.389 | 2.571 |
| CPB duration | 0.395 | 2.529 |
| MHCA temperature | 0.82 | 1.22 |
| POD2Lac | 0.809 | 1.237 |
| LnPOD2sMb | 0.273 | 3.66 |
| ΔCr | 0.479 | 2.087 |
| LnPOD2CK_MB | 0.515 | 1.94 |

38.Collinearity Statistics of LnPOD2sMb+Model^b^+ΔCr+LnPOD2CysC with any AKI Regression Analysis

|  | Collinearity Statistics | |
| --- | --- | --- |
|  | Tolerance | VIF |
| Male | 0.901 | 1.11 |
| Age | 0.822 | 1.216 |
| BMI | 0.799 | 1.252 |
| Hypertension | 0.869 | 1.15 |
| Pre-eGFR | 0.7 | 1.429 |
| Pre-WBC | 0.851 | 1.175 |
| Surgery duration | 0.387 | 2.583 |
| CPB duration | 0.395 | 2.535 |
| MHCA temperature | 0.82 | 1.22 |
| POD2Lac | 0.82 | 1.22 |
| LnPOD2sMb | 0.4 | 2.502 |
| ΔCr | 0.331 | 3.021 |
| LnPOD2CysC | 0.406 | 2.464 |

39.Collinearity Statistics of LnPOD2sMb+Model^b^ with Severe AKI Regression Analysis

|  | Collinearity Statistics | |
| --- | --- | --- |
|  | Tolerance | VIF |
| Male | 0.905 | 1.105 |
| Age | 0.845 | 1.184 |
| BMI | 0.812 | 1.232 |
| Hypertension | 0.896 | 1.116 |
| Pre-eGFR | 0.825 | 1.212 |
| Pre-WBC | 0.852 | 1.174 |
| Surgery duration | 0.389 | 2.57 |
| CPB duration | 0.396 | 2.527 |
| MHCA temperature | 0.821 | 1.218 |
| POD2Lac | 0.842 | 1.188 |
| LnPOD2sMb | 0.62 | 1.613 |

40.Collinearity Statistics of LnPOD2sMb+Model^b^+ΔCr with Severe AKI Regression Analysis

|  | Collinearity Statistics | |
| --- | --- | --- |
|  | Tolerance | VIF |
| Male | 0.905 | 1.105 |
| Age | 0.844 | 1.185 |
| BMI | 0.799 | 1.252 |
| Hypertension | 0.889 | 1.125 |
| Pre-eGFR | 0.798 | 1.253 |
| Pre-WBC | 0.852 | 1.174 |
| Surgery duration | 0.389 | 2.57 |
| CPB duration | 0.396 | 2.528 |
| MHCA temperature | 0.82 | 1.219 |
| POD2Lac | 0.829 | 1.206 |
| LnPOD2sMb | 0.403 | 2.484 |
| ΔCr | 0.491 | 2.036 |

41.Collinearity Statistics of LnPOD2sMb+Model^b^+ΔCr+LnPOD2BNP with Severe AKI Regression Analysis

|  | Collinearity Statistics | |
| --- | --- | --- |
|  | Tolerance | VIF |
| Male | 0.891 | 1.122 |
| Age | 0.842 | 1.187 |
| BMI | 0.785 | 1.274 |
| Hypertension | 0.878 | 1.139 |
| Pre-eGFR | 0.797 | 1.254 |
| Pre-WBC | 0.85 | 1.176 |
| Surgery duration | 0.387 | 2.581 |
| CPB duration | 0.39 | 2.564 |
| MHCA temperature | 0.803 | 1.245 |
| POD2Lac | 0.823 | 1.215 |
| LnPOD2sMb | 0.392 | 2.553 |
| ΔCr | 0.489 | 2.043 |
| LnPOD2BNP | 0.836 | 1.196 |

42.Collinearity Statistics of LnPOD2sMb+Model^b^+ΔCr+LnPOD2cTnI with Severe AKI Regression Analysis

|  | Collinearity Statistics | |
| --- | --- | --- |
|  | Tolerance | VIF |
| Male | 0.898 | 1.114 |
| Age | 0.844 | 1.185 |
| BMI | 0.792 | 1.263 |
| Hypertension | 0.882 | 1.133 |
| Pre-eGFR | 0.798 | 1.253 |
| Pre-WBC | 0.83 | 1.205 |
| Surgery duration | 0.389 | 2.57 |
| CPB duration | 0.391 | 2.56 |
| MHCA temperature | 0.82 | 1.219 |
| POD2Lac | 0.821 | 1.217 |
| LnPOD2sMb | 0.335 | 2.982 |
| ΔCr | 0.488 | 2.051 |
| LnPOD2cTnI | 0.508 | 1.968 |

43.Collinearity Statistics of LnPOD2sMb+Model^b^+ΔCr+LnPOD2CK_MB with Severe AKI Regression Analysis

|  | Collinearity Statistics | |
| --- | --- | --- |
|  | Tolerance | VIF |
| Male | 0.901 | 1.11 |
| Age | 0.844 | 1.185 |
| BMI | 0.746 | 1.341 |
| Hypertension | 0.886 | 1.129 |
| Pre-eGFR | 0.79 | 1.267 |
| Pre-WBC | 0.849 | 1.178 |
| Surgery duration | 0.389 | 2.571 |
| CPB duration | 0.395 | 2.529 |
| MHCA temperature | 0.82 | 1.22 |
| POD2Lac | 0.809 | 1.237 |
| LnPOD2sMb | 0.273 | 3.66 |
| ΔCr | 0.479 | 2.087 |
| LnPOD2CK_MB | 0.515 | 1.94 |

44.Collinearity Statistics of LnPOD2sMb+Model^b^+ΔCr+LnPOD2CysC with Severe AKI Regression Analysis

|  | Collinearity Statistics | |
| --- | --- | --- |
|  | Tolerance | VIF |
| Male | 0.901 | 1.11 |
| Age | 0.844 | 1.185 |
| BMI | 0.746 | 1.341 |
| Hypertension | 0.886 | 1.129 |
| Pre-eGFR | 0.79 | 1.267 |
| Pre-WBC | 0.849 | 1.178 |
| Surgery duration | 0.389 | 2.571 |
| CPB duration | 0.395 | 2.529 |
| MHCA temperature | 0.82 | 1.22 |
| POD2Lac | 0.809 | 1.237 |
| LnPOD2sMb | 0.273 | 3.66 |
| ΔCr | 0.479 | 2.087 |
| LnPOD2CysC | 0.515 | 1.94 |

45.Collinearity Statistics of LnPOD2sMb+Model^b^ with 30-day Mortality Regression Analysis

|  | Collinearity Statistics | |
| --- | --- | --- |
|  | Tolerance | VIF |
| Male | 0.905 | 1.105 |
| Age | 0.845 | 1.184 |
| BMI | 0.812 | 1.232 |
| Hypertension | 0.896 | 1.116 |
| Pre-eGFR | 0.825 | 1.212 |
| Pre-WBC | 0.852 | 1.174 |
| Surgery duration | 0.389 | 2.57 |
| CPB duration | 0.396 | 2.527 |
| MHCA temperature | 0.821 | 1.218 |
| POD2Lac | 0.842 | 1.188 |
| LnPOD2sMb | 0.62 | 1.613 |

46.Collinearity Statistics of LnPOD2sMb+Model^b^+ΔCr with 30-day Mortality Regression Analysis

|  | Collinearity Statistics | |
| --- | --- | --- |
|  | Tolerance | VIF |
| Male | 0.905 | 1.105 |
| Age | 0.844 | 1.185 |
| BMI | 0.799 | 1.252 |
| Hypertension | 0.889 | 1.125 |
| Pre-eGFR | 0.798 | 1.253 |
| Pre-WBC | 0.852 | 1.174 |
| Surgery duration | 0.389 | 2.57 |
| CPB duration | 0.396 | 2.528 |
| MHCA temperature | 0.82 | 1.219 |
| POD2Lac | 0.829 | 1.206 |
| LnPOD2sMb | 0.403 | 2.484 |
| ΔCr | 0.491 | 2.036 |

47.Collinearity Statistics of LnPOD2sMb+Model^b^+ΔCr+LnPOD2BNP with 30-day Mortality Regression Analysis

|  | Collinearity Statistics | |
| --- | --- | --- |
|  | Tolerance | VIF |
| Male | 0.891 | 1.122 |
| Age | 0.842 | 1.187 |
| BMI | 0.785 | 1.274 |
| Hypertension | 0.878 | 1.139 |
| Pre-eGFR | 0.797 | 1.254 |
| Pre-WBC | 0.85 | 1.176 |
| Surgery duration | 0.387 | 2.581 |
| CPB duration | 0.39 | 2.564 |
| MHCA temperature | 0.803 | 1.245 |
| POD2Lac | 0.823 | 1.215 |
| LnPOD2sMb | 0.392 | 2.553 |
| ΔCr | 0.489 | 2.043 |
| LnPOD2BNP | 0.836 | 1.196 |

48.Collinearity Statistics of LnPOD2sMb+Model^b^+ΔCr+LnPOD2cTnI with 30-day Mortality Regression Analysis

|  | Collinearity Statistics | |
| --- | --- | --- |
|  | Tolerance | VIF |
| Male | 0.898 | 1.114 |
| Age | 0.844 | 1.185 |
| BMI | 0.792 | 1.263 |
| Hypertension | 0.882 | 1.133 |
| Pre-eGFR | 0.798 | 1.253 |
| Pre-WBC | 0.83 | 1.205 |
| Surgery duration | 0.389 | 2.57 |
| CPB duration | 0.391 | 2.56 |
| MHCA temperature | 0.82 | 1.219 |
| POD2Lac | 0.821 | 1.217 |
| LnPOD2sMb | 0.335 | 2.982 |
| ΔCr | 0.488 | 2.051 |
| LnPOD2cTnI | 0.508 | 1.968 |

49.Collinearity Statistics of LnPOD2sMb+Model^b^+ΔCr+LnPOD2CK_MB with 30-day Mortality Regression Analysis

|  | Collinearity Statistics | |
| --- | --- | --- |
|  | Tolerance | VIF |
| Male | 0.901 | 1.11 |
| Age | 0.844 | 1.185 |
| BMI | 0.746 | 1.341 |
| Hypertension | 0.886 | 1.129 |
| Pre-eGFR | 0.79 | 1.267 |
| Pre-WBC | 0.849 | 1.178 |
| Surgery duration | 0.389 | 2.571 |
| CPB duration | 0.395 | 2.529 |
| MHCA temperature | 0.82 | 1.22 |
| POD2Lac | 0.809 | 1.237 |
| LnPOD2sMb | 0.273 | 3.66 |
| ΔCr | 0.479 | 2.087 |
| LnPOD2CK_MB | 0.515 | 1.94 |

50.Collinearity Statistics of LnPOD2sMb+Model^b^+ΔCr+LnPOD2CysC with 30-day Mortality Regression Analysis

|  | Collinearity Statistics | |
| --- | --- | --- |
|  | Tolerance | VIF |
| Male | 0.901 | 1.11 |
| Age | 0.822 | 1.216 |
| BMI | 0.799 | 1.252 |
| Hypertension | 0.869 | 1.15 |
| Pre-eGFR | 0.7 | 1.429 |
| Pre-WBC | 0.851 | 1.175 |
| Surgery duration | 0.387 | 2.583 |
| CPB duration | 0.395 | 2.535 |
| MHCA temperature | 0.82 | 1.22 |
| POD2Lac | 0.82 | 1.22 |
| LnPOD2sMb | 0.4 | 2.502 |
| ΔCr | 0.331 | 3.021 |
| LnPOD2CysC | 0.406 | 2.464 |

51.Collinearity Statistics of LnPOD3sMb+Model^b^ with any AKI Regression Analysis

|  | Collinearity Statistics | |
| --- | --- | --- |
|  | Tolerance | VIF |
| Male | 0.908 | 1.101 |
| Age | 0.833 | 1.2 |
| BMI | 0.795 | 1.258 |
| Hypertension | 0.895 | 1.118 |
| Pre-eGFR | 0.838 | 1.193 |
| Pre-WBC | 0.861 | 1.161 |
| Surgery duration | 0.4 | 2.5 |
| CPB duration | 0.395 | 2.529 |
| MHCA temperature | 0.833 | 1.2 |
| POD3Lac | 0.878 | 1.138 |
| LnPOD3sMb | 0.685 | 1.461 |

52.Collinearity Statistics of LnPOD3sMb+Model^b^+ΔCr with any AKI Regression Analysis

|  | Collinearity Statistics | |
| --- | --- | --- |
|  | Tolerance | VIF |
| Male | 0.906 | 1.104 |
| Age | 0.833 | 1.2 |
| BMI | 0.784 | 1.276 |
| Hypertension | 0.891 | 1.123 |
| Pre-eGFR | 0.823 | 1.215 |
| Pre-WBC | 0.861 | 1.162 |
| Surgery duration | 0.399 | 2.505 |
| CPB duration | 0.395 | 2.534 |
| MHCA temperature | 0.832 | 1.202 |
| POD3Lac | 0.789 | 1.267 |
| LnPOD3sMb | 0.504 | 1.984 |
| ΔCr | 0.511 | 1.956 |

53.Collinearity Statistics of LnPOD3sMb+Model^b^+ΔCr+LnPOD3BNP with any AKI Regression Analysis

|  | Collinearity Statistics | |
| --- | --- | --- |
|  | Tolerance | VIF |
| Male | 0.885 | 1.13 |
| Age | 0.83 | 1.205 |
| BMI | 0.777 | 1.288 |
| Hypertension | 0.883 | 1.133 |
| Pre-eGFR | 0.823 | 1.215 |
| Pre-WBC | 0.859 | 1.164 |
| Surgery duration | 0.398 | 2.514 |
| CPB duration | 0.391 | 2.558 |
| MHCA temperature | 0.81 | 1.235 |
| POD3Lac | 0.787 | 1.27 |
| LnPOD3sMb | 0.489 | 2.043 |
| ΔCr | 0.507 | 1.972 |
| LnPOD3BNP | 0.806 | 1.24 |

54.Collinearity Statistics of LnPOD3sMb+Model^b^+ΔCr+LnPOD3cTnI with any AKI Regression Analysis

|  | Collinearity Statistics | |
| --- | --- | --- |
|  | Tolerance | VIF |
| Male | 0.904 | 1.106 |
| Age | 0.833 | 1.2 |
| BMI | 0.776 | 1.289 |
| Hypertension | 0.89 | 1.124 |
| Pre-eGFR | 0.822 | 1.216 |
| Pre-WBC | 0.85 | 1.177 |
| Surgery duration | 0.399 | 2.505 |
| CPB duration | 0.389 | 2.571 |
| MHCA temperature | 0.829 | 1.207 |
| POD3Lac | 0.758 | 1.319 |
| LnPOD3sMb | 0.413 | 2.422 |
| ΔCr | 0.507 | 1.972 |
| LnPOD3cTnI | 0.52 | 1.922 |

55.Collinearity Statistics of LnPOD3sMb+Model^b^+ΔCr+LnPOD3CK_MB with any AKI Regression Analysis

|  | Collinearity Statistics | |
| --- | --- | --- |
|  | Tolerance | VIF |
| Male | 0.876 | 1.141 |
| Age | 0.833 | 1.201 |
| BMI | 0.756 | 1.323 |
| Hypertension | 0.891 | 1.123 |
| Pre-eGFR | 0.823 | 1.216 |
| Pre-WBC | 0.856 | 1.169 |
| Surgery duration | 0.399 | 2.509 |
| CPB duration | 0.391 | 2.561 |
| MHCA temperature | 0.831 | 1.204 |
| POD3Lac | 0.779 | 1.284 |
| LnPOD3sMb | 0.315 | 3.176 |
| ΔCr | 0.508 | 1.968 |
| LnPOD3CK_MB | 0.488 | 2.048 |

56.Collinearity Statistics of LnPOD3sMb+Model^b^+ΔCr+LnPOD3CysC with any AKI Regression Analysis

|  | Collinearity Statistics | |
| --- | --- | --- |
|  | Tolerance | VIF |
| Male | 0.899 | 1.112 |
| Age | 0.829 | 1.207 |
| BMI | 0.784 | 1.276 |
| Hypertension | 0.871 | 1.149 |
| Pre-eGFR | 0.65 | 1.537 |
| Pre-WBC | 0.859 | 1.164 |
| Surgery duration | 0.399 | 2.509 |
| CPB duration | 0.395 | 2.535 |
| MHCA temperature | 0.829 | 1.206 |
| POD3Lac | 0.754 | 1.327 |
| LnPOD3sMb | 0.478 | 2.094 |
| ΔCr | 0.335 | 2.982 |
| LnPOD3CysC | 0.353 | 2.833 |

57.Collinearity Statistics of LnPOD3sMb+Model^b^ with Severe AKI Regression Analysis

|  | Collinearity Statistics | |
| --- | --- | --- |
|  | Tolerance | VIF |
| Male | 0.908 | 1.101 |
| Age | 0.833 | 1.2 |
| BMI | 0.795 | 1.258 |
| Hypertension | 0.895 | 1.118 |
| Pre-eGFR | 0.838 | 1.193 |
| Pre-WBC | 0.861 | 1.161 |
| Surgery duration | 0.4 | 2.5 |
| CPB duration | 0.395 | 2.529 |
| MHCA temperature | 0.833 | 1.2 |
| POD3Lac | 0.878 | 1.138 |
| LnPOD3sMb | 0.685 | 1.461 |

58.Collinearity Statistics of LnPOD3sMb+Model^b^+ΔCr with Severe AKI Regression Analysis

|  | Collinearity Statistics | |
| --- | --- | --- |
|  | Tolerance | VIF |
| Male | 0.906 | 1.104 |
| Age | 0.833 | 1.2 |
| BMI | 0.784 | 1.276 |
| Hypertension | 0.891 | 1.123 |
| Pre-eGFR | 0.823 | 1.215 |
| Pre-WBC | 0.861 | 1.162 |
| Surgery duration | 0.399 | 2.505 |
| CPB duration | 0.395 | 2.534 |
| MHCA temperature | 0.832 | 1.202 |
| POD3Lac | 0.789 | 1.267 |
| LnPOD3sMb | 0.504 | 1.984 |
| ΔCr | 0.511 | 1.956 |

59.Collinearity Statistics of LnPOD3sMb+Model^b^+ΔCr+LnPOD3BNP with Severe AKI Regression Analysis

|  | Collinearity Statistics | |
| --- | --- | --- |
|  | Tolerance | VIF |
| Male | 0.885 | 1.13 |
| Age | 0.83 | 1.205 |
| BMI | 0.777 | 1.288 |
| Hypertension | 0.883 | 1.133 |
| Pre-eGFR | 0.823 | 1.215 |
| Pre-WBC | 0.859 | 1.164 |
| Surgery duration | 0.398 | 2.514 |
| CPB duration | 0.391 | 2.558 |
| MHCA temperature | 0.81 | 1.235 |
| POD3Lac | 0.787 | 1.27 |
| LnPOD3sMb | 0.489 | 2.043 |
| ΔCr | 0.507 | 1.972 |
| LnPOD3BNP | 0.806 | 1.24 |

60.Collinearity Statistics of LnPOD3sMb+Model^b^+ΔCr+LnPOD3cTnI with Severe AKI Regression Analysis

|  | Collinearity Statistics | |
| --- | --- | --- |
|  | Tolerance | VIF |
| Male | 0.904 | 1.106 |
| Age | 0.833 | 1.2 |
| BMI | 0.776 | 1.289 |
| Hypertension | 0.89 | 1.124 |
| Pre-eGFR | 0.822 | 1.216 |
| Pre-WBC | 0.85 | 1.177 |
| Surgery duration | 0.399 | 2.505 |
| CPB duration | 0.389 | 2.571 |
| MHCA temperature | 0.829 | 1.207 |
| POD3Lac | 0.758 | 1.319 |
| LnPOD3sMb | 0.413 | 2.422 |
| ΔCr | 0.507 | 1.972 |
| LnPOD3cTnI | 0.52 | 1.922 |

61.Collinearity Statistics of LnPOD3sMb+Model^b^+ΔCr+LnPOD3CK_MB with Severe AKI Regression Analysis

|  | Collinearity Statistics | |
| --- | --- | --- |
|  | Tolerance | VIF |
| Male | 0.876 | 1.141 |
| Age | 0.833 | 1.201 |
| BMI | 0.756 | 1.323 |
| Hypertension | 0.891 | 1.123 |
| Pre-eGFR | 0.823 | 1.216 |
| Pre-WBC | 0.856 | 1.169 |
| Surgery duration | 0.399 | 2.509 |
| CPB duration | 0.391 | 2.561 |
| MHCA temperature | 0.831 | 1.204 |
| POD3Lac | 0.779 | 1.284 |
| LnPOD3sMb | 0.315 | 3.176 |
| ΔCr | 0.508 | 1.968 |
| LnPOD3CK_MB | 0.488 | 2.048 |

62.Collinearity Statistics of LnPOD3sMb+Model^b^+ΔCr+LnPOD3CysC with Severe AKI Regression Analysis

|  | Collinearity Statistics | |
| --- | --- | --- |
|  | Tolerance | VIF |
| Male | 0.899 | 1.112 |
| Age | 0.829 | 1.207 |
| BMI | 0.784 | 1.276 |
| Hypertension | 0.871 | 1.149 |
| Pre-eGFR | 0.65 | 1.537 |
| Pre-WBC | 0.859 | 1.164 |
| Surgery duration | 0.399 | 2.509 |
| CPB duration | 0.395 | 2.535 |
| MHCA temperature | 0.829 | 1.206 |
| POD3Lac | 0.754 | 1.327 |
| LnPOD3sMb | 0.478 | 2.094 |
| ΔCr | 0.335 | 2.982 |
| LnPOD3CysC | 0.353 | 2.833 |

63.Collinearity Statistics of LnPOD3sMb+Model^b^ with 30-day Mortality Regression Analysis

|  | Collinearity Statistics | |
| --- | --- | --- |
|  | Tolerance | VIF |
| Male | 0.908 | 1.101 |
| Age | 0.833 | 1.2 |
| BMI | 0.795 | 1.258 |
| Hypertension | 0.895 | 1.118 |
| Pre-eGFR | 0.838 | 1.193 |
| Pre-WBC | 0.861 | 1.161 |
| Surgery duration | 0.4 | 2.5 |
| CPB duration | 0.395 | 2.529 |
| MHCA temperature | 0.833 | 1.2 |
| POD3Lac | 0.878 | 1.138 |
| LnPOD3sMb | 0.685 | 1.461 |

64.Collinearity Statistics of LnPOD3sMb+Model^b^+ΔCr with 30-day Mortality Regression Analysis

|  | Collinearity Statistics | |
| --- | --- | --- |
|  | Tolerance | VIF |
| Male | 0.906 | 1.104 |
| Age | 0.833 | 1.2 |
| BMI | 0.784 | 1.276 |
| Hypertension | 0.891 | 1.123 |
| Pre-eGFR | 0.823 | 1.215 |
| Pre-WBC | 0.861 | 1.162 |
| Surgery duration | 0.399 | 2.505 |
| CPB duration | 0.395 | 2.534 |
| MHCA temperature | 0.832 | 1.202 |
| POD3Lac | 0.789 | 1.267 |
| LnPOD3sMb | 0.504 | 1.984 |
| ΔCr | 0.511 | 1.956 |

65.Collinearity Statistics of LnPOD3sMb+Model^b^+ΔCr+LnPOD3BNP with 30-day Mortality Regression Analysis

|  | Collinearity Statistics | |
| --- | --- | --- |
|  | Tolerance | VIF |
| Male | 0.885 | 1.13 |
| Age | 0.83 | 1.205 |
| BMI | 0.777 | 1.288 |
| Hypertension | 0.883 | 1.133 |
| Pre-eGFR | 0.823 | 1.215 |
| Pre-WBC | 0.859 | 1.164 |
| Surgery duration | 0.398 | 2.514 |
| CPB duration | 0.391 | 2.558 |
| MHCA temperature | 0.81 | 1.235 |
| POD3Lac | 0.787 | 1.27 |
| LnPOD3sMb | 0.489 | 2.043 |
| ΔCr | 0.507 | 1.972 |
| LnPOD3BNP | 0.806 | 1.24 |

66.Collinearity Statistics of LnPOD3sMb+Model^b^+ΔCr+LnPOD3cTnI with 30-day Mortality Regression Analysis

|  | Collinearity Statistics | |
| --- | --- | --- |
|  | Tolerance | VIF |
| Male | 0.904 | 1.106 |
| Age | 0.833 | 1.2 |
| BMI | 0.776 | 1.289 |
| Hypertension | 0.89 | 1.124 |
| Pre-eGFR | 0.822 | 1.216 |
| Pre-WBC | 0.85 | 1.177 |
| Surgery duration | 0.399 | 2.505 |
| CPB duration | 0.389 | 2.571 |
| MHCA temperature | 0.829 | 1.207 |
| POD3Lac | 0.758 | 1.319 |
| LnPOD3sMb | 0.413 | 2.422 |
| ΔCr | 0.507 | 1.972 |
| LnPOD3cTnI | 0.52 | 1.922 |

67.Collinearity Statistics of LnPOD3sMb+Model^b^+ΔCr+LnPOD3CK_MB with 30-day Mortality Regression Analysis

|  | Collinearity Statistics | |
| --- | --- | --- |
|  | Tolerance | VIF |
| Male | 0.876 | 1.141 |
| Age | 0.833 | 1.201 |
| BMI | 0.756 | 1.323 |
| Hypertension | 0.891 | 1.123 |
| Pre-eGFR | 0.823 | 1.216 |
| Pre-WBC | 0.856 | 1.169 |
| Surgery duration | 0.399 | 2.509 |
| CPB duration | 0.391 | 2.561 |
| MHCA temperature | 0.831 | 1.204 |
| POD3Lac | 0.779 | 1.284 |
| LnPOD3sMb | 0.315 | 3.176 |
| ΔCr | 0.508 | 1.968 |
| LnPOD3CK_MB | 0.488 | 2.048 |

68.Collinearity Statistics of LnPOD3sMb+Model^b^+ΔCr+LnPOD3CysC with 30-day Mortality Regression Analysis

|  | Collinearity Statistics | |
| --- | --- | --- |
|  | Tolerance | VIF |
| Male | 0.899 | 1.112 |
| Age | 0.829 | 1.207 |
| BMI | 0.784 | 1.276 |
| Hypertension | 0.871 | 1.149 |
| Pre-eGFR | 0.65 | 1.537 |
| Pre-WBC | 0.859 | 1.164 |
| Surgery duration | 0.399 | 2.509 |
| CPB duration | 0.395 | 2.535 |
| MHCA temperature | 0.829 | 1.206 |
| POD3Lac | 0.754 | 1.327 |
| LnPOD3sMb | 0.478 | 2.094 |
| ΔCr | 0.335 | 2.982 |
| LnPOD3CysC | 0.353 | 2.833 |
