# Supplementary material for: Serum Myoglobin Is Associated With Postoperative Acute Kidney Injury in Stanford Type A Aortic Dissection
Source: Front Med (Lausanne). 2022 Feb 22;9:821418. doi: 10.3389/fmed.2022.821418 (PMC8902311; doi:10.3389/fmed.2022.821418)
Supplement: Supplementary file 6 [file Table_6.DOCX]

Table E6.1. Prediction performance of the POD1 Ln(NT-proBNP)

|  | Any AKI(95%IC) | Severe AKI(95%IC) | 30-day mortality(95%IC) |
| --- | --- | --- | --- |
| AUC(95%IC) |  |  |  |
| Model^b^ | 0.75(0.71,0.79) | 0.79(0.75,0.83) | 0.74(0.70,0.78) |
| Model^b^+Ln(NT-proBNP) | 0.77(0.73,0.81) | 0.83(0.79,0.86) | 0.76(0.72,0.80) |
| ΔAUC | 0.02(-0.01,0.04) | 0.03(-0.01,0.02) | 0.02(-0.02,0.05) |
| Continuous NRI(95%) |  |  |  |
| All NRI | 0.40^**^(0.20,0.61) | 0.28^*^(0.08,0.48) | 0.28^*^(0.08,0.48) |
| IDI | 0.03^*^(0.01,0.05) | 0.03^*^(0.02,0.04) | 0.03^*^(0.02,0.05) |

a: Any AKI is KDIGO stage 1 or higher. Severe AKI is KDIGO stage 2&3.

b: Modelb: age, sex, BMI, hypertension, preoperative eGFR and preoperative WBC, surgery duration, cardiopulmonary bypass duration, the lowest rectal temperature

c: *P-value<0.05; **P-value<0.001

Table E6.2. Prediction performance of the POD1 Ln(cTnI)

|  | Any AKI(95%IC) | Severe AKI(95%IC) | 30-day mortality(95%IC) |
| --- | --- | --- | --- |
| AUC(95%IC) |  |  |  |
| Model^b^ | 0.75(0.71,0.79) | 0.79(0.75,0.83) | 0.74(0.70,0.78) |
| Model^b^+Ln(cTnI) | 0.78(0.74,0.82) | 0.81(0.76,0.84) | 0.76(0.71,0.80) |
| ΔAUC | 0.03(<0.01,0.06)^*^ | 0.01(-0.01,0.03) | 0.01(-0.01,0.04) |
| Continuous NRI(95%) |  |  |  |
| All NRI | 0.30^**^(0.10,0.51) | 0.29^*^(0.09,0.49) | 0.19(-0.02,0.39) |
| IDI | 0.04^*^(0.02,0.06) | 0.02^*^(0.01,0.04) | 0.03^*^(<0.01,0.01) |

a: Any AKI is KDIGO stage 1 or higher. Severe AKI is KDIGO stage 2&3.

b: Modelb: age, sex, BMI, hypertension, preoperative eGFR and preoperative WBC, surgery duration, cardiopulmonary bypass duration, the lowest rectal temperature

c: *P-value<0.05; **P-value<0.001

Table E6.3. Prediction performance of the POD1 Ln(CK-MB)

| CK-MB | Any AKI(95%IC) | Severe AKI(95%IC) | 30-day mortality(95%IC) |
| --- | --- | --- | --- |
| AUC(95%IC) |  |  |  |
| Model^b^ | 0.75(0.71,0.79) | 0.79(0.75,0.83) | 0.74(0.70,0.78) |
| Model^b^+Ln(CK-MB) | 0.77(0.73,0.81) | 0.80(0.76,0.84) | 0.77(0.72,0.81) |
| ΔAUC | 0.02(-0.01,0.04) | 0.01(-0.01,0.02) | 0.02(-0.01,0.05) |
| Continuous NRI(95%) |  |  |  |
| All NRI | 0.20(-0.01,0.41) | 0.20(-0.01,0.41) | 0.19(-0.02,0.39) |
| IDI | 0.02^*^(0.01,0.04) | 0.01^*^(<0.01,0.02) | 0.01^*^(0.01,0.02) |

a: Any AKI is KDIGO stage 1 or higher. Severe AKI is KDIGO stage 2&3.

b: Modelb: age, sex, BMI, hypertension, preoperative eGFR and preoperative WBC, surgery duration, cardiopulmonary bypass duration, the lowest rectal temperature

c: *P-value<0.05; **P-value<0.001

Table E6.4. Prediction performance of the POD1 Ln(CysC)

| CysC | Any AKI(95%IC) | Severe AKI(95%IC) | 30-day mortality(95%IC) |
| --- | --- | --- | --- |
| AUC(95%IC) |  |  |  |
| Model^b^ | 0.75(0.71,0.79) | 0.79(0.75,0.83) | 0.74(0.70,0.78) |
| Model^b^+Ln(CysC) | 0.88(0.85,0.91) | 0.86(0.82,0.89) | 0.77(0.72,0.81) |
| ΔAUC | 0.13(0.09,0.18)^**^ | 0.06(0.02,0.11)^*^ | 0.02(-0.02,0.07) |
| Continuous NRI(95%) |  |  |  |
| All NRI | 0.96(0.77,1.14)^**^ | 0.64(0.47,0.81)^**^ | 0.75(0.58,0.92)^**^ |
| IDI | 0.14(0.10,0.18)^**^ | 0.03(0.02,0.05)^**^ | 0.03(0.02,0.04)^**^ |

a: Any AKI is KDIGO stage 1 or higher. Severe AKI is KDIGO stage 2&3.

b: Modelb: age, sex, BMI, hypertension, preoperative eGFR and preoperative WBC, surgery duration, cardiopulmonary bypass duration, the lowest rectal temperature

c: *P-value<0.05; **P-value<0.001
