# Supplementary figures and images for: Serum Myoglobin Is Associated With Postoperative Acute Kidney Injury in Stanford Type A Aortic Dissection
Source: Front Med (Lausanne). 2022 Feb 22;9:821418. doi: 10.3389/fmed.2022.821418 (PMC8902311; doi:10.3389/fmed.2022.821418)

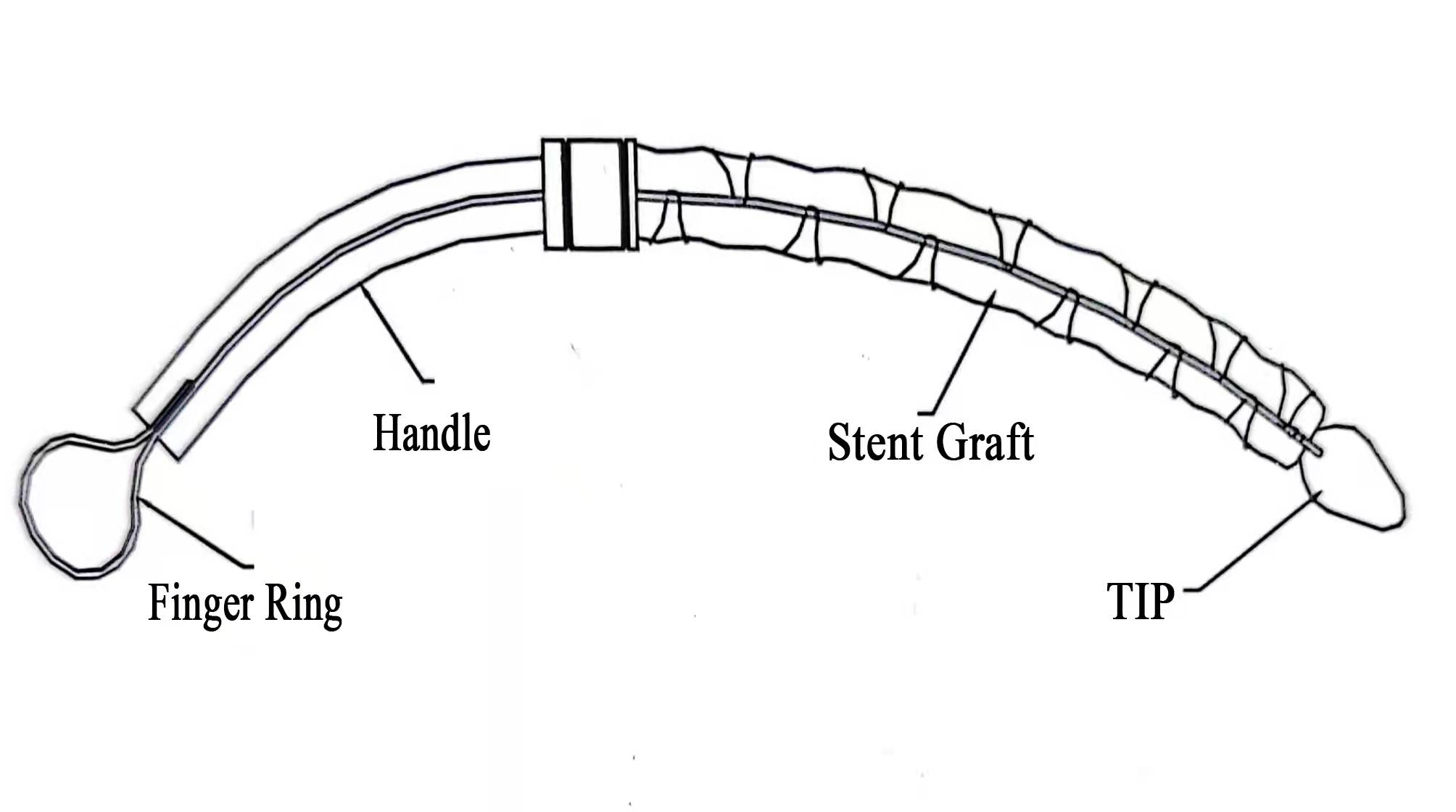

Supplement: Supplementary Figure 1 — Flow chart of study exclusion and inclusion. TAAD, Type A aortic dissection; TAR, total arch replacement; FET, frozen elephant trunk; ESRD: AKI: acute kidney injury. [file Image_1.TIF]

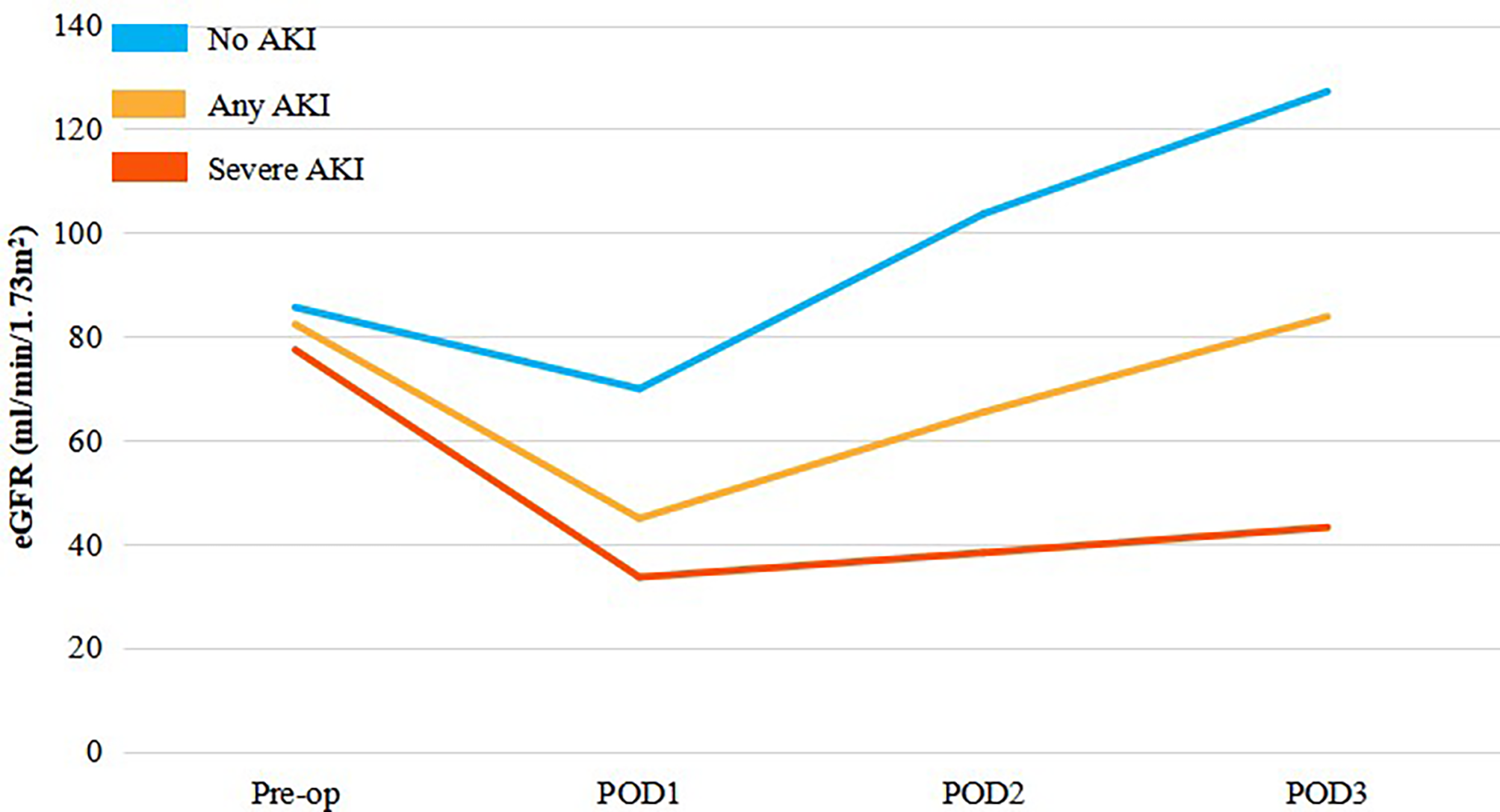

Supplement: Supplementary Figure 2 — Frozen elephant trunk (MicroPort Medical, Shanghai, China). [file Image_2.TIF]

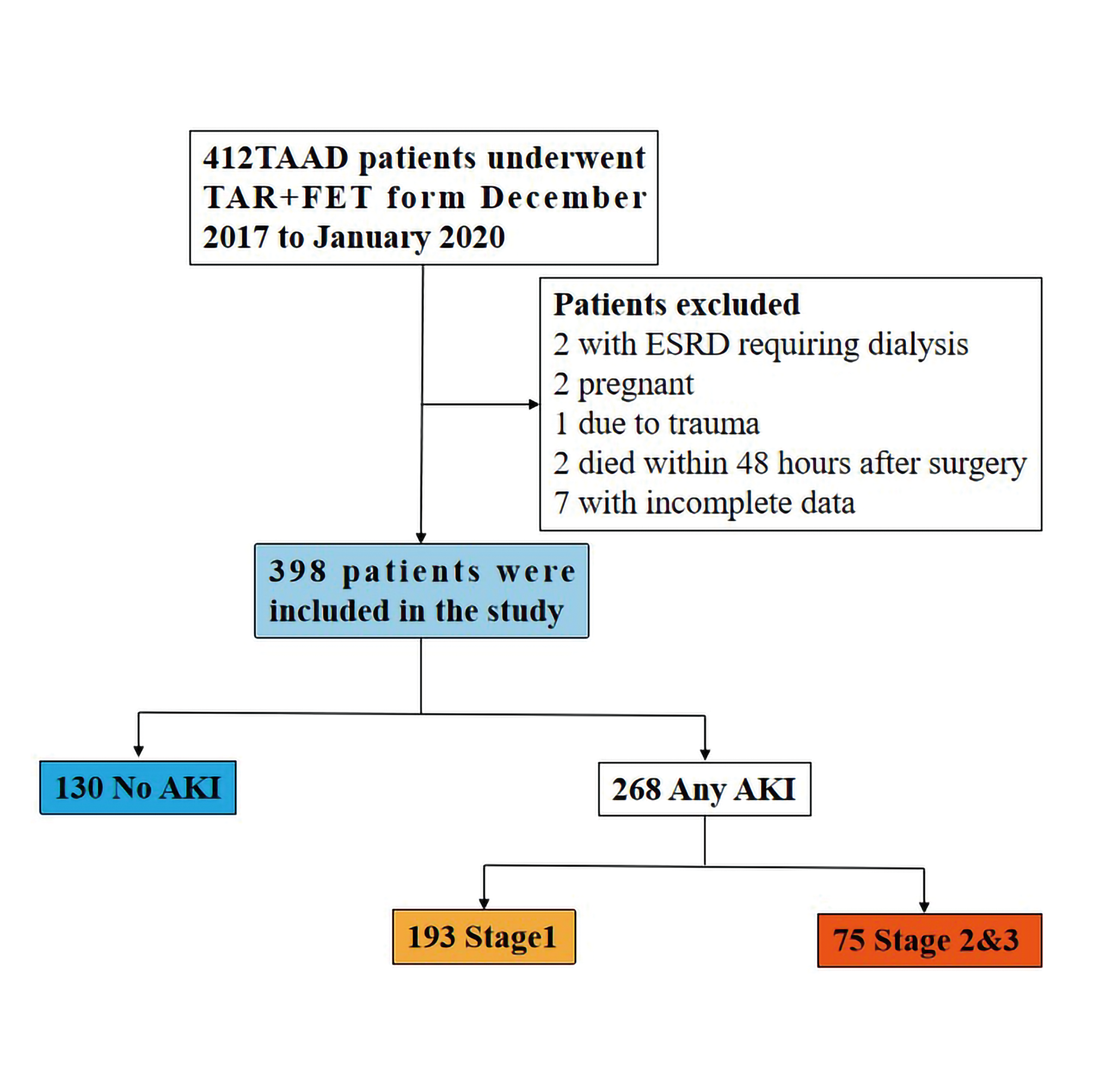

Supplement: Supplementary Figure 3 — Daily changes in eGFR. [file Image_3.TIF]

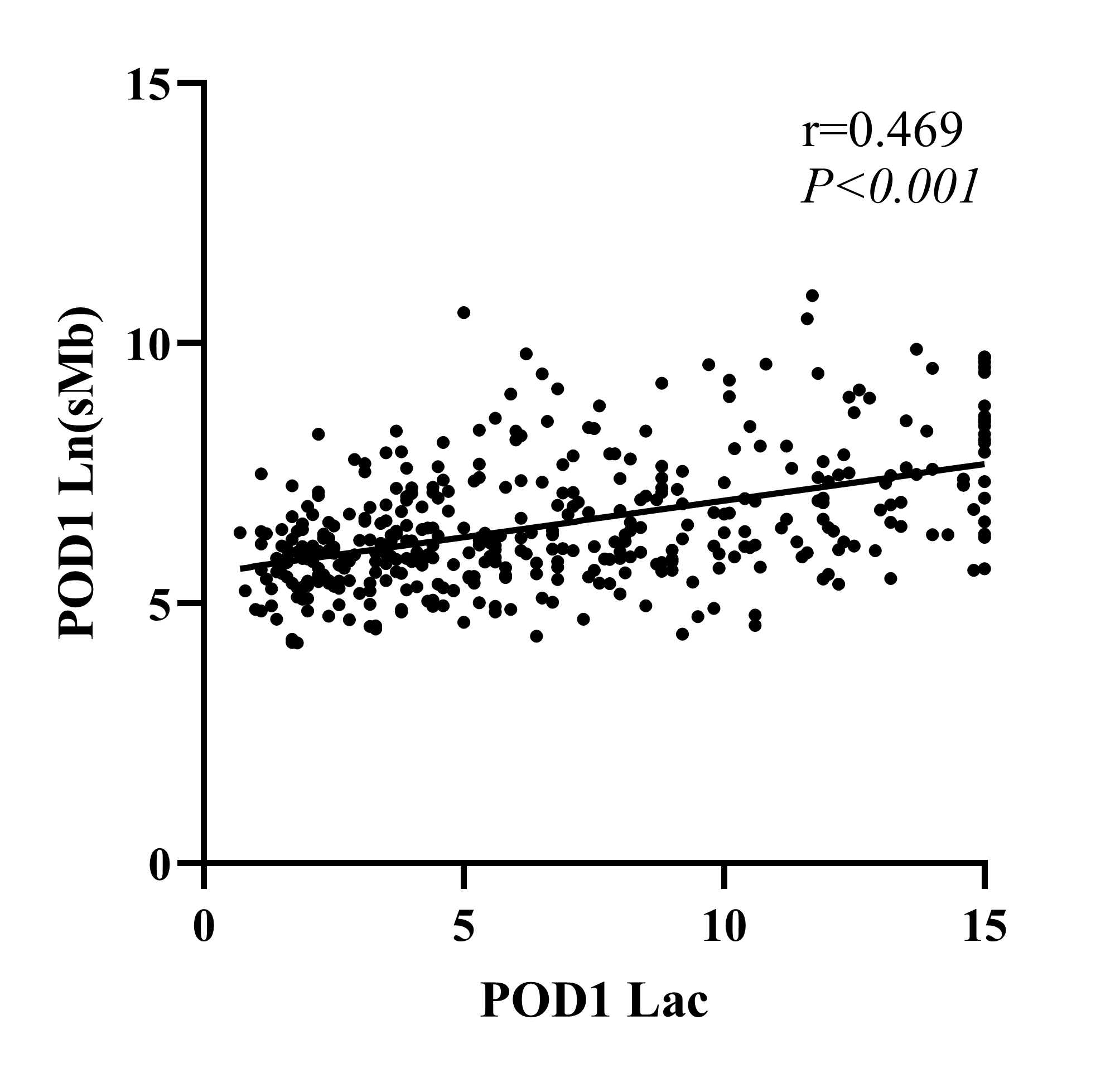

Supplement: Supplementary Figure 4 — Correlation between lactate and Ln(sMb) on POD1. [file Image_4.TIF]
